# Heterogeneous Catalytic Ozonation of Pharmaceuticals: Optimization of the Process by Response Surface Methodology

**DOI:** 10.3390/nano14211747

**Published:** 2024-10-30

**Authors:** Nikoletta Tsiarta, Wolfgang Gernjak, Hrvoje Cajner, Gordana Matijašić, Lidija Ćurković

**Affiliations:** 1 Catalan Institute of Water Research (ICRA)-CERCA, Carrer Emili Grahit 101, 17003 Girona, Spain; ntsiarta@icra.cat; 2 Faculty of Sciences, University of Girona, Campus de Montilivi, 17003 Girona, Spain; 3 Faculty of Mechanical Engineering and Naval Architecture, University of Zagreb, Ivana Lučića 5, 10002 Zagreb, Croatia; hrvoje.cajner@fsb.unizg.hr; 4 Catalan Institution for Research and Advanced Studies (ICREA), 08010 Barcelona, Spain; 5 Faculty of Chemical Engineering and Technology, University of Zagreb, 10000 Zagreb, Croatia; gmatijas@fkit.unizg.hr

**Keywords:** heterogeneous catalytic ozonation, pharmaceuticals, hydroxyl radicals, response surface methodology

## Abstract

Batch heterogeneous catalytic ozonation experiments were performed using commercial and synthesized nanoparticles as catalysts in aqueous ozone. The transferred ozone dose (TOD) ranged from 0 to 150 μM, and nanoparticles were added in concentrations between 0 and 1.5 g L^−1^, with all experiments conducted at 20 °C and a total volume of 240 mL. A Ce-doped TiO_2_ catalyst (1% molar ratio of Ce/Ti) was synthesized via the sol–gel method. Response surface methodology (RSM) was applied to identify the most significant factors affecting the removal of selected pharmaceuticals, with TOD emerging as the most critical variable. Higher TOD resulted in greater removal efficiencies. Furthermore, it was found that the commercially available metal oxides α-Al_2_O_3_, Mn_2_O_3_, TiO_2_, and CeO_2_, as well as the synthesized CeTiO_x_, did not increase the catalytic activity of ozone during the degradation of ibuprofen (IBF) and para-chlorobenzoic acid (pCBA). Carbamazepine (CBZ) and diclofenac (DCF) are compounds susceptible to ozone oxidation, thus their complete degradation at 150 μM transferred ozone dose was attained. The limited catalytic effect was attributed to the rapid consumption of ozone within the first minute of reaction, as well as the saturation of catalyst active sites by water molecules, which inhibited effective ozone adsorption and subsequent hydroxyl radical generation (^●^OH).

## 1. Introduction

Organic micropollutants (OMPs), also referred to as contaminants of emerging concern (CECs), cover a wide range of substances, including pharmaceuticals, personal care products, detergents, and pesticides. These compounds are frequently detected in wastewater streams at very low concentrations (μg L^−1^ or ng L^−1^). When inadequately treated in both household and industrial wastewater, OMPs pose a significant risk to aquatic ecosystems, with far-reaching implications for human health [1]. As water covers 70% of our planet’s surface, freshwater bodies are especially vulnerable to these compounds, which exist in both soluble and insoluble forms.

Wastewater contains a mixture of organic and inorganic constituents, either dissolved or in suspension. The fate of these pollutants depends largely on the self-cleaning capacity of the receiving water bodies, where natural biodegradation can play an essential role. However, when the influx of pollutants exceeds this capacity, contamination occurs, leading to water quality degradation [2]. This problem is intensified by the widespread use of chemicals in various human activities and industries, which ultimately end up in water bodies, altering their physical, chemical, and biological characteristics [3]. To mitigate these effects and protect the environment, wastewater must undergo thorough treatment before its release.

Conventional wastewater treatment plants (WWTPs), combining physicochemical and biological processes, are designed to reduce nutrient and organic matter loads in compliance with environmental regulations, such as the EU Water Framework Directive 2000/60/EC [4]. These processes help control eutrophication and excessive oxygen consumption in water bodies when reclaimed water is disposed into them. However, numerous studies have shown that even high-performance WWTPs struggle to fully eliminate OMPs due to their recalcitrant nature [5,6]. In particular, it is well-documented that recalcitrant OMPs, including antibiotics, corrosion inhibitors, artificial sweeteners, chelating agents, and perfluorinated compounds, among others, exhibit significant resistance to conventional treatment processes [7].

One promising solution for the removal of these persistent compounds is ozone-based treatment systems. Ozone (O_3_), a strong oxidant, has shown significant potential in degrading OMPs, achieving both direct oxidation and the generation of hydroxyl radicals (^●^OH) that can mineralize complex organic structures [8,9]. For example, ozone can directly degrade OMPs, often achieving mineralization depending on factors like ozone contact time and concentration. Alternatively, O_3_ oxidation generates highly effective radicals that facilitate chemical oxidation, effectively breaking down a broad spectrum of compounds, including pharmaceuticals and antibiotics [9,10,11].

Its application has grown over the past two decades, especially in industrial wastewater treatment, gradually replacing traditional chlorine-based processes due to its superior disinfection capacity and lower energy requirements. It is also a predominant oxidation technology, praised for its cost-effectiveness and energy efficiency compared to other advanced oxidation techniques, such as UV disinfection [12,13]. In particular, the catalytic ozonation process—where ozone interacts with metal oxide catalysts—enhances the removal efficiency of recalcitrant OMPs, as the catalysts promote the formation of reactive oxygen species, such as hydroxyl radicals (^●^OH), and improve overall degradation kinetics.

Heterogeneous catalytic ozonation represents a particularly promising advanced oxidation process (AOP). By using solid-phase catalysts, such as metal oxides, the process can achieve significant pollutant mineralization at ambient temperature [14], while preventing catalyst degradation. This method has shown substantial improvements in organic matter oxidation compared to conventional ozonation [15,16,17]. Moreover, by combining catalytic ozonation with response surface methodology (RSM), it is possible to optimize the process conditions for the removal of pharmaceuticals. The chosen model compounds are carbamazepine (CBZ), diclofenac (DCF), and ibuprofen (IBP), which are often found in wastewater streams and are notoriously resistant to traditional treatment methods [18]. *Para*-chlorobenzoic acid (pCBA) was also used in the study as an O_3_/^●^OH probe compound [19]. While previous studies have explored catalytic ozonation systems for AOPs [20,21,22,23,24], few have applied RSM to heterogeneous catalytic ozonation, which forms the novelty of this work.

In this study, we explore the potential of heterogeneous catalytic ozonation as an effective strategy for the removal of recalcitrant OMPs from wastewater, offering insights into its mechanisms and application. The nanoparticles α-Al_2_O_3_, Mn_2_O_3_, TiO_2_ (P25), CeO_2_, and CeTiO_x_ were selected as catalysts for their demonstrated ability to enhance the degradation of OMPs, including pharmaceuticals and recalcitrant compounds. These metal oxides have been shown to facilitate the decomposition of O_3_ and promote ^●^OH generation, leading to enhanced OMPs degradation. For instance, aloumina has been found to facilitate pCBA [24] and diethyl phthalate [25] degradation. Manganese oxides have been found to significantly improved the removal of oxalic acid [26], ibuprofen [27], and toluene [28]. Furthermore, titania oxides in ozonation systems, have demonstrated efficacy in the oxidation of carbamazepine [29], aspartame [30], and parabens [31]. CeO_2_ and CeTiO_x_, with their redox-active surfaces, further enhance ozone activation, leading to improved degradation of bisphenol [32], DEET [33], and ciprofloxacin [34]. These catalysts, when used in catalytic ozonation systems, offer a robust approach for removing recalcitrant pollutants from wastewater. The RSM was ultimately used to determine the optimum parameters for the catalytic ozonation of the selected OMPs and the interaction between the studied parameters.

## 2. Materials and Methods

### 2.1. Chemicals and Materials

Carbamazepine (CBZ, 99.8%, C_15_H_12_N_2_O, CAS: 298-46-4), ibuprofen (IBP, 98.9% C_13_H_18_O_2_, CAS: 15687-27-1;), diclofenac sodium salt (DCF, 98%, C_14_H_10_C_l2_NNaO_2_, CAS: 15307-79-6), and *para*-chlorobenzoic acid (pCBA, 99%, C_7_H_5_ClO_2_, CAS: 74-11-3). CBZ, IBP, and DCF were purchased from Sigma-Aldrich (St. Louis, MO, USA), whereas the pCBA was purchased from ACROS Organics (Waltham, MA, USA). Table 1 summarizes the characteristics of those compounds. Sodium bicarbonate (NaHCO_3_, CAS: 144-55-8) used as a buffer was obtained from Sigma-Aldrich. All commercial metal oxides or nanoparticles (NPs), alpha-aluminum oxide (α-Al_2_O_3_, 78 nm, CAS: 1344-28-1), manganese oxide (Mn_2_O_3_, 28 nm, CAS: 1317-34-6), and ceria oxide (CeO_2_, 8–28 nm, CAS:1306-38-3) were purchased by Nanografi (Nanografi Nanotechnology, Ankara, Turkey). Titanium oxide (TiO_2_, P25, CAS: 13463-67-7) was purchased from Sigma-Aldrich. For the synthesis of the ceria doped titania (CeTiO_x_), the following chemicals were used: Potassium indigotrisulfonate (indigo, C_16_H_7_K_3_N_2_O_11_S_3_, CAS: 67627-18-3) was purchased from Sigma Aldrich and was used to determine ozone concentration. Acetonitrile gradient grade for liquid chromatography (ACN, C_2_H_3_N, CAS: 75-05-8) was obtained from Merck Millipore (Burlington, MA, USA).

### 2.2. Synthesis of CeTiOx Nanoparticles

The ceria-doped titania (CeTiO_x_) nanoparticles were synthesized using the sol–gel method [35]. To prepare the precursor solution, metal nitrate salt (CeN_3_O_9_·6H_2_O) and titania isopropoxide 97% (C_12_H_28_O_4_Ti) were dissolved in a mixture of isopropanol, acetylacetone, and nitric acid. The Ce to Ti molar ratio was 1% [33]. The solution was stirred overnight, then coated onto glass plates and placed in the oven at 80 °C for 9 h. Subsequently, the temperature was raised to 120 °C, and the solution was left in the oven for an additional 6 h. After the aging and drying process, the samples were brought to room temperature and introduced into a furnace to eliminate impurities and facilitate crystal powder formation. The furnace was set to 450 °C for 2 h. Finally, the resulting solids were collected in a mortar and pulverized into a fine powder using a pestle. A detailed scheme is provided in the Supplementary Material (Figure S1).

### 2.3. Characterization of the Nanoparticles

The electrical charge on the particles’ surface in a liquid medium, the zeta potential, was determined with a Zetasizer Ultra (Malvern Panalytical, Malvern, UK). A mass of 0.1 g of the sample was suspended in 100 mL of deionized water and subjected to ultrasonication for 20 min. The pH of the suspension was adjusted to between 2 and 12 using either HCl or NaOH (0.1 mol L^−1^). Before each measurement, the suspensions were ultrasonically treated for 30 s. The pH values of the solutions were determined using a digital pH meter from Mettler Toledo (Columbus, OH, USA).

The ASAP 2000 instrument (Micromeritics Corporation in Norcross, GA, USA) was used to determine the BET-specific surface area, pore volume, and pore size distribution based on nitrogen adsorption and desorption isotherms data. Before analysis, samples were degassed at 120 °C for 10 h until a vacuum of 50 µm Hg was reached to remove all physically adsorbed substances. Samples were placed in tubes with a 10 cm^3^ bulb and a 1/2″ stem. Data were collected in the range of 0 to 1 relative pressure (p/p0). A total of 40 adsorption/desorption points were collected after each equilibration interval of 5 s. The Barret–Joyner–Halenda model was used to calculate the pore size distribution of the sample using data from the adsorption and desorption branches of the nitrogen isotherms. The BET surface area was calculated using five points in the range of p/p0 from 0.05 to 0.24.

The characterization of the commercially available nanoparticles is presented in Table S1 (Supplementary Material) as provided by the manufacturer. On the other hand, the elemental analysis of CeTiOx was carried out using the powder X-ray diffraction method (PXRD) with a Shimadzu XRD 6000. PXRD patterns were generated using a D8 Advance X-ray diffractometer (Bruker, Billerica, MA, USA). The X-ray diffractometer utilized Cu Kα radiation with an acceleration voltage of 30 kV and a current of 30 mA, following the Bragg–Brentano focusing geometry. The analysis was conducted in a step-scan mode with a 0.02° 2θ step size, covering a 2θ range of 10–80°, with a counting time of 0.6 s. The analysis of the PXRD diffractogram was executed using the HighScore Plus software (Malvern Panalytical, Malvern, UK) package.

Additionally, the Fourier transform infrared spectroscopy (FTIR) analysis of the synthesized CeTiO_x_ sample was performed using a spectrometer (IRSpirit, Shimadzu, Tokyo, Japan) equipped with an ATR (Attenuated Total Reflectance) accessory. The energy-dispersive X-ray microscopy spectrum (EDS) of the CeTiO_x_ sample was collected using the Nano Esprit 2 detector (Bruker, Billerica, MA, USA) at 10 kV and 15 mm working distance at 1000 magnification within the Vega Easyprobe 3 electron microscope (Tescan, Brno, Czech Republic).

### 2.4. Response Surface Methodology

The surface response graphs for evaluating the different treatments were generated using the Design Expert software “Version 13.0.0” (Stat-Ease, Inc., Minneapolis, MN, USA). The Design of Experiments (DoE) approach was used to create an empirical mathematical model that can forecast the result of a dependent variable in relation to a set of independent variables. The outcomes from the degradation experiments were acquired by employing a mixed-level factorial design, wherein three independent variables (factors) were systematically adjusted across multiple levels (as detailed in Table 2). A total of 80 experiments were carried out using a randomized design to ensure unbiased results. These experiments covered all possible combinations of the selected variables: transferred ozone dose (TOD), nanoparticle’s type, and nanoparticle dose (NPs), allowing for a thorough exploration of the parameter space. The randomized approach also minimized experimental errors and provided robust data for response surface methodology (RSM) analysis. The percentage (%) removal of each of the four model compounds was used as the response variable.

The analysis of significance was conducted using the Analysis of Variance (ANOVA) method, employing a significance threshold of 5%, a threshold commonly used to classify the statistical significance of the evaluated statistical properties of the model. Regression models were calculated and graphically represented through response surface plots, estimating the regression coefficients.

### 2.5. Batch Experiments with Nanoparticles

**Figure 1:**
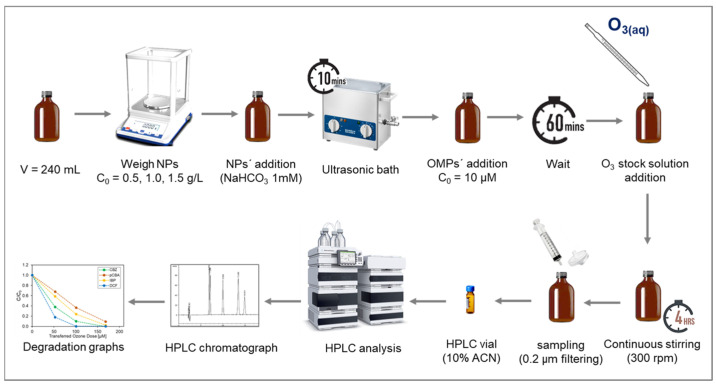
Schematic representation of the batch experiment procedure.

Batch experiments were performed in amber bottles of 240 mL working volume with different doses of nanoparticles and aqueous ozone (O_3(aq)_). The matrix consisted of demineralized water and bicarbonate buffer at 1 mM to keep pH stable at 7.4–7.6. All experiments were conducted at room temperature using a laboratory-scale ozone reactor (ANSEROS, COM-AD-04, Tübingen, Germany). Ozone gas was continuously injected into a 3 L glass bottle filled with deionized water (in-house production) to achieve a concentrated stock solution of dissolved ozone (approx. 45 mg L^−1^). The initial concentration of the micropollutants was 10 μM. The transferred ozone doses (TOD) ranged from 0, 50, 100, and 150 μM (0−7.2 mg L^−1^). The O_3_(l) stock solution concentration was determined spectrophotometrically at 260 nm (Shimadzu UV-1800, Shimadzu Corporation, Kyoto, Japan), and the corresponding volume was added to each reaction bottle to achieve the required TOD. The catalytic activity of the α-Al_2_O_3_, Mn_2_O_3_, CeO_2_, TiO_2_, and CeTiOx with different particle sizes was examined using three different concentrations: 0, 0.5, 1.0, and 1.5 mg L^−1^. The 240 mL bottles were left to react at ambient temperature for four hours after ozone addition (Figure 1). Samples were collected, filtrated through a 0.22 μM nylon filters (Whatman, Merck, Darmstadt, Germany), quenched with thiosulfate (Na_2_S_2_O_3_) [36], and finally analyzed in the HPLC.

### 2.6. Ozone Degradation Studies

To examine the ozone decomposition in different matrices, batch experiments similar to batch experiments with nanoparticles were performed (see Section 2.5). The ozonation procedure was kept the same, but different components were added to the amber bottles. The highest ozone dose (150 μM) was used to quantify the ozone residual concentration over time. Two different OMPs’ concentrations of 2 μM and 10 μM and the presence or absence of 1 g L^−1^ CeTiOx were examined. In addition, the effect of the bicarbonate on ozone depletion was investigated by adding or not the buffer in the treated matrix. The residual ozone concentration was determined by the indigo method [37], adding 4 mL of the sampling volume into the volumetric flask containing the 0.5 M phosphate buffer and the 1 mM indigo solution. The sampling points were set to every half minute for the first 3 min and then every five minutes until 20 min, ending with samplings at 40 min and 60 min. Indigo solution absorbance was spectrophotometrically measured at 600 nm. An indigo Vs ozone calibration curve (Figure S2) was constructed, and the absorption values were converted into concentrations. Figure S3 depicts the calibration curves for the model compounds.

### 2.7. Analytical Methods

The model micropollutants were identified and quantified by high-performance liquid chromatography (HPLC) in reversed-phase using a liquid chromatography instrument (HPLC-UV Agilent 1200) coupled to a quaternary pump and equipped with an ultraviolet-visible detector and an autosampler (both from Agilent Technologies, Santa Clara, CA, USA). Chromatographic separation was performed using a C18 column (Microsorb-MV 100-5 250 × 4.6 mm) at a working temperature of 30 °C and flow of 0.8 mL L^−1^. The samples were directly injected through the autosampler with an injection volume of 200 μL. The effluent was a combination of ACN, milli-Q water, and 0.3% of formic acid with 10% of ACN. The gradient varied over time (40 min), and more information regarding the analytical method is provided in the Supplementary Material (Section 4). The lower limit of quantification for all OMPs was 0.25 ± 0.16 μM (54.69 ± 32.80 μg L^−1^).

## 3. Results and Discussion

The catalytic activity of different metal oxides/nanoparticles (NPs), commercial and synthesized, towards ozone was examined by comparing the degradation of recalcitrant to ozone compounds, such as ibuprofen and pCBA, during different treatments. The nanoparticles used in the catalytic ozonation experiments were the commercial α-Al_2_O_3_, Mn_2_O_3_, CeO_2_, TiO_2_, and the synthesized CeTiO_x_. The characterization techniques used to identify their catalytic potential properties, as well as the degradation graphs, are listed below.

### 3.1. Characterization of the Nanoparticles

**Figure 2:**
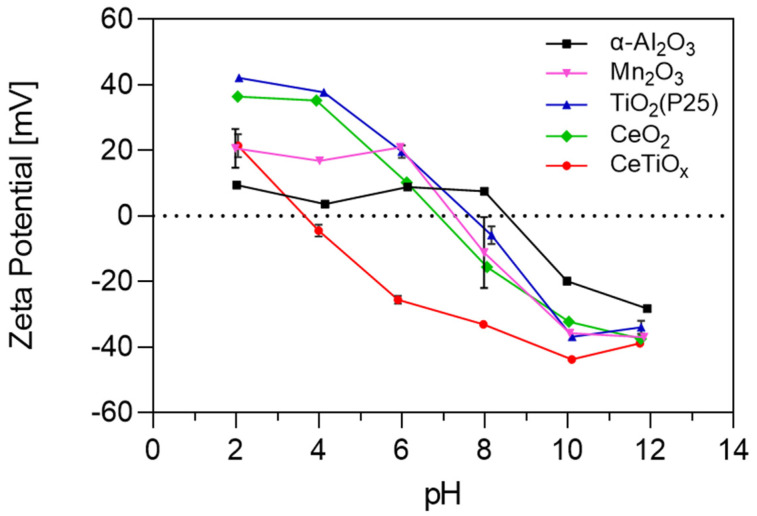
The zeta potential of five different nanoparticles as a function of pH.

In catalysis, the zeta potential of nanoparticles can influence their catalytic activity. For example, specific reactions may be facilitated or hindered by the surface charge of catalyst nanoparticles. Studying the zeta potential can help identify which nanoparticles are more suitable for specific catalytic applications. The zeta potential analysis presented in Figure 2 offers significant insights into the surface properties of the five NPs. The graph illustrates how the zeta potential of the selected nanoparticles varies with changes in pH. This is a critical aspect of nanoparticle behavior, as it influences their interactions with reactants and, thus, their catalytic performance.

TiO_2_, Mn_2_O_3_, and CeO_2_ exhibit similar pH-dependent trends, all starting as positively charged in acidic pH and transitioning to negatively charged in basic environments. This similarity suggests they may share similar surface chemistry or reactivity under certain conditions. However, Mn_2_O_3_ showed a less positive charge than TiO_2_ and CeO_2_. This implies that Mn_2_O_3_ may not attract negatively charged molecules as strongly. The point of zero charge (PZC) for TiO_2_, Mn_2_O_3_, and CeO_2_ is 7.7, 7.2, and 6.9, respectively, suggesting that the surface of the particles was slightly negative to neutral under the experimental conditions. Due to the addition of bicarbonate as a buffer, the pH of the working solutions was maintained stable at 7.6–7.8.

α-Al_2_O_3_ also displays a pH-dependent charge, with a positive charge until pH 8. However, it is noted as being less positively charged and less negatively charged in acidic and basic pH, respectively, compared to all other nanoparticles. This difference implies that it has a milder or less intense surface charge, suggesting not very strong electrostatic repulsion or attraction forces with other particles or molecules, and that it is more likely to aggregate or flocculate. Exhibiting the higher PZC at pH 8.6, it is the only nanoparticle that remains positively charged in the working solution. On the other hand, CeTiO_x_ (synthesized nanoparticle) stands out as having a consistently negatively charged surface across a wide pH range, from 3 to 12. Notably, CeTiO_x_ showcases a zeta potential value that tends to repel each other more strongly, leading to better colloidal stability. This distinctive behavior suggests CeTiO_x_ has unique catalytic characteristics, especially when other nanoparticles might be positively charged. Similar to Lee et al. [33], CeTiO_x_ exhibited good catalytic performance under ozonation for the degradation of DEET: 80% removal after 10 min.

At the working pH, the state of the metal oxides was positive for α-Al_2_O_3_ and TiO_2_ and negative for Mn_2_O_3_, CeO_2_, and the synthesized CeTiO_x_. This can be explained by the relation of the PZC to pH (Equations (1)–(3)). The catalyst’s surface becomes positively charged when the PZC exceeds the solution’s pH. Lower solution pH strengthens protonation, reducing the nucleophilicity of the hydroxyl groups on the catalyst’s surface. Subsequently, this hinders their interaction with ozone, resulting in decreased catalytic activity. On the other hand, when the PZC is lower than the pH of the solution, the catalyst’s surface becomes negatively charged, attracting O_3_ binding and radical ^●^OH generation. In some cases, though it was reported that the catalyst sites were more active, reaching the maximum of their catalytic activity when the catalyst was uncharged, neither protonated nor deprotonated [24,38,39].

(1) $$pH<{pH}_{PZC}:M-OH+H^{+}↔{{M-OH}_{2}}^{+}(positivelycharged)$$

(2) $$pH={pH}_{PZC}:M-OH+H_{2}O(neutral)$$

(3) $$pH>{pH}_{PZC}:M-OH+{OH}^{-}↔{M-O}^{-}+H_{2}O(negativelycharged)$$

**Figure 3:**
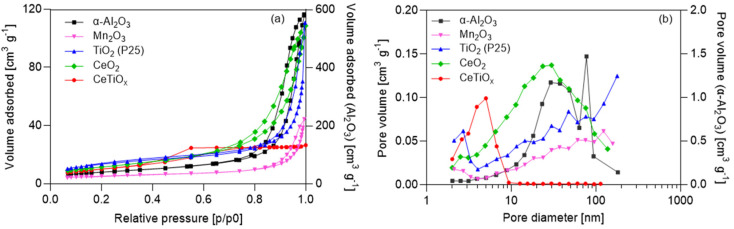
(**a**) Nitrogen adsorption-desorption isotherms, volume adsorbed as a function of the relative pressure, and (**b**) pore volume distribution of the nanoparticles.

Furthermore, understanding the pore size distribution of a catalyst is crucial in various chemical and catalytic processes, especially in the field of heterogeneous catalysis. Pores in catalyst materials play a significant role in determining their performance and efficiency. A well-known technique used for assessing the pore characteristics of catalysts is BET (Brunauer–Emmett–Teller). The tested nanoparticles were analyzed, and the nitrogen adsorption–desorption isotherms, as well as the pore size distribution graphs, were constructed (Figure 3).

The data show that α-Al_2_O_3_ exhibited the highest values regarding the specific area (m^2^ g^−1^) and pore volume (cm^3^ g^−1^). In addition, the isotherms are one order of magnitude higher than the rest of the nanoparticles (the second y-axis indicates the values for α-Al_2_O_3_). On the other hand, the Mn_2_O_3_ specific area was significantly lower than the other NPs. Regarding the specific area, the nanoparticles can be classified as α-Al_2_O_3_ > TiO_2_ > CeO_2_ > Mn_2_O_3_ > CeTiO_x_, and regarding pore volume as α-Al_2_O_3_ > TiO_2_ > CeO_2_ > CeTiO_x_ > Mn_2_O_3_. This suggests that α-Al_2_O_3_ has more active sites for catalysis and is a good candidate for catalytic ozonation. Its disadvantage, however, lies in the fact that α-Al_2_O_3_ is mostly a macropore material (<50 nm). Macroporous materials may be advantageous if the catalytic ozonation process involves larger reactant molecules or if mass transfer limitations are a significant consideration. It is well-documented, however, that mesoporous materials (2–50 nm) have shown good performance in catalytic ozonation due to their structure [16,40,41]. They often have high surface areas, providing a large number of active sites for catalytic reactions [15,40].

According to the pore size distribution data (Table 3 and Figure 3b), CeTiO_x_ could be characterized as a mesoporous material, with most pores smaller than 10 nm. There is a certain amount of micropores, but not large enough to show a significant volume of micropores. According to the new IUPAC classification [42], there are eight types of isotherms, six of which were identified in the 1985 IUPAC Manual on Reporting Physisorption Data for Gas/Solid Systems. The type IV (a) isotherm is characteristic of mesoporous materials, and this is consistent with all materials (α-Al_2_O_3_, Mn_2_O_3_, TiO_2_, CeO_2_, and CeTiO_x_). The presence of adsorption hysteresis in type IV (a) isotherms (Figure 3a) is expected and is related to capillary condensation and evaporation in the mesopores. In addition, the shape of the adsorption hysteresis loop correlates with the pore size distribution, pore geometry, and pore connectivity.

Additionally, the H_3_ hysteresis loop shown for the α-Al_2_O_3_, Mn_2_O_3_, TiO_2_, and CeO_2_ samples is observed in materials with a pore network consisting of macropores that are not entirely filled with pore condensate. Also, the pore size distribution of all samples showed the presence of macropores, i.e., pores larger than 50 nm. On the other hand, the H_2_(a) hysteresis loop obtained for the CeTiO_x_ sample displays the presence of bottlenecks in the pore shape. Therefore, the desorption branch of the isotherm can be attributed to the blocking/percolation due to the narrow bottlenecks.

The decrease in surface area, pore volume, and pore size distribution observed in CeTiO_x_ compared to the other nanoparticles, as shown in Table 3, can be attributed to the structural changes caused by the incorporation of Ce ions into the TiO_2_. Ce-doping is known to modify the surface characteristics and crystal structure of TiO_2_, potentially leading to a reduction in surface area due to particle agglomeration and changes in pore structure. Specifically, CeTiO_x_ exhibited a relatively lower specific surface area (36.4 m^2^ g^−1^) compared to pure TiO_2_ (49 m^2^ g^−1^), and its mean pore diameter was also smaller (4.5 nm). These changes suggest that Ce-doping may have led to the formation of narrower pores (bottlenecks), as indicated by the H2(a) hysteresis loop (Figure 3a), which is characteristic of materials with pore-blocking or percolation mechanisms. This structural alteration can hinder gas diffusion into the deeper layers of the material, reducing the number of active sites available for catalytic reactions.

Furthermore, the lower pore volume (0.043 cm^3^ g^−1^) of CeTiO_x_ compared to TiO_2_ (0.169 cm^3^ g^−1^) might also indicate a decrease in porosity due to Ce incorporation. This observation aligns with the reduced catalytic performance of CeTiO_x_ in some experiments, as its limited surface area and pore structure may restrict the adsorption and activation of ozone and pollutants during catalytic ozonation. Despite these limitations, the CeTiO_x_ sample retains mesoporous characteristics with most pores smaller than 10 nm, which is beneficial for specific catalytic processes, though these material property changes impact its efficiency in this specific context.

**Figure 4:**
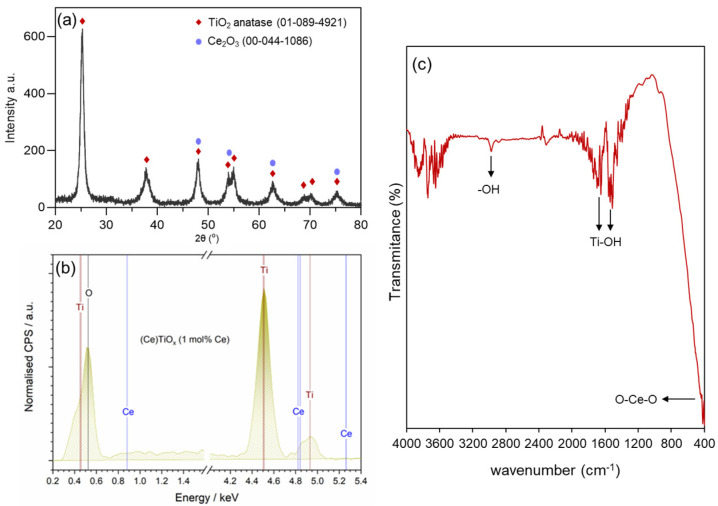
(**a**) PXRD pattern, (**b**) energy-dispersive X-ray (EDS) spectrum, and (**c**) FTIR spectrum of CeTiO_x_ sample.

The observed reduction in CeTiO_x_ characteristics is further supported by its crystalline structure. These textural changes often accompany modifications in the material’s crystallinity, which can be examined through X-ray diffraction analysis. The powder X-ray diffraction (PXRD) pattern of CeTiO_x_ synthesized at 450 °C, represented in Figure 4a, provides further insight into how the Ce doping affected the crystalline phase of TiO_2_. The sample exhibited seven distinguished diffraction peaks at 25°, 38°, 48°, 54°, 63°, and 70°. According to the XRD spectra, all diffraction peaks of the synthesized material corresponded to TiO_2_ anatase (ICSD 01-89-4921). In addition, no impurity peaks were observed, indicating that the Ce ions were effectively doped without causing any changes to the crystal structure of TiO_2_ [33]. For the calculations of crystal size, the spherical shape of crystallite was assumed [43], and the peaks in the diffractogram were fitted using the Lorentzian function. The average crystal size of the CeTiO_x_ was calculated at 10.3 ± 1.0 nm, and a similar size was reported by Lee and his collaborators [33]. The calculations were based on the Scherrer formula below [44].

(4) $$D=\frac{K\;\lambda }{FWHM\;cos\theta }$$

where D is the mean size of the crystallite; *K* is a shape constant (0.89); *λ* is the X-ray wavelength at 0.154 nm; *FWHM* is the full width at half-maximum of the diffraction peak; and *θ* is the Bragg angle.

To further confirm the successful incorporation of the Ce into the unit cell of TiO_2_ structure (interstitial doping), energy-dispersive spectroscopy (EDS) was used. In Figure 4b, it can be clearly seen that the elements Ti, Ce, and O were present in the CeTiO_x_ sample. Moreover, the FTIR spectrum of the synthesized TiO_2_ (Figure 4c) showed distinct Ti-OH vibrational stretches around 1500 and 1600 cm^−1^, alongside asymmetric and symmetric –OH stretches near 2900 cm^−1^, which are likely due to adsorbed water. A notable O-Ce-O vibration is also detected at 415 cm^−1^. These findings align well with the previously reported literature [45,46].

Additionally, in recent research conducted by one of our co-authors [34], it was demonstrated that small amounts of Ce (up to 0.08 wt.%) can be successfully incorporated into the TiO_2_ lattice through wet chemistry synthesis. This method promoted a homogeneous pre-crystalline network, facilitating the substitutional doping of Ce^4+^ ions (with a crystal radius of 0.092 nm) in place of Ti^4+^ ions (0.065 nm) within the TiO_2_ unit cell. This doping alters the unit cell parameters, as confirmed by XRD analysis. Beyond a certain threshold, excess Ce remains outside the lattice, forming separate cerium oxide phases. Our synthesis procedure followed the same methodology, ensuring the successful incorporation of Ce in the TiO_2_ lattice, consistent with the literature [33,34], and is further supported by our XRD measurements.

### 3.2. Effect of Matrix on Ozone Depletion

**Figure 5:**
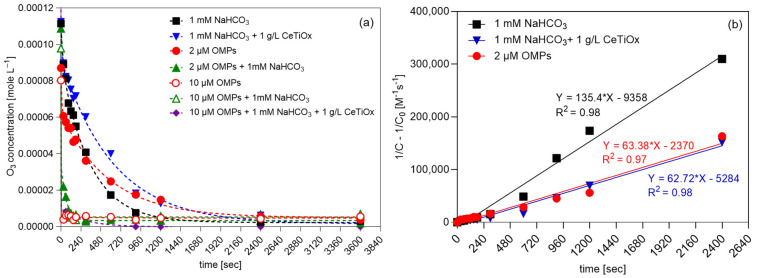
(**a**) Degradation of ozone over time and (**b**) second-order kinetics plots of ozone in different matrices (TOD = 100 μM, 240 mL total volume, [NaHCO_3_] = 1 mM, [OMPs] = 2 or 10 μM, catalyst concentration = 1 g^−1^).

The ozone depletion in different matrices was examined to understand better the exposure time of the OMPs to ozone and how the presence of bicarbonate (HCO_3_^–^) and catalyst affect ozone decomposition. All tests were performed at room temperature, and a theoretical TOD of 100 μM of ozone was used. Figure 5a displays the degradation of ozone (mole L^−1^) over time (s). The ozone was present for at least 20 min after its addition to the solutions containing only (i) 1 mM NaHCO_3_, (ii) 1 mM NaHCO_3_ and catalyst (1 g L^−1^ CeTiO_x_), and (iii) the lowest concentration of OMPs, i.e., 2 μM in demineralized water. On the other hand, the rest of the tested matrices immediately reacted with ozone, and a sudden drop in its concentration was observed within the first 30 s. In the Supplementary Material (Figure S4), it is apparent that lower OMP concentration slightly lessened the ozone decomposition; 20% of the initial ozone dose was present after 1 min. In the other cases, less than 5% of the initial ozone was present after 1 min of reaction.

The working concentration of OMPs at 10 μM in the presence of bicarbonate decreased the ozone concentration quickly, implying that the exposure of the OMPs’ mixture to ozone was limited to the first minute of the reaction. Similarly, the 10 μM OMP solution in demineralized water decreased ozone immediately. Thus, it can be concluded that the bicarbonate effect on ozone decomposition is negligible when the organic load is high (Figure S4b) as organic compounds intensify the ozone depletion. However, when the organic load is low (2 μM), the presence of bicarbonate leads to faster decomposition (Figure S4a), and this can be supported by the fact that bicarbonate is competing for hydroxyl radicals (^●^OH) produced by ozone decomposition. It is reported that bicarbonate has a scavenging effect towards ^●^OH, producing bicarbonate radicals (HCO_3_^●–^) and intermediates that quench the radical chain carrier [47,48]. Moreover, it can be observed that the presence of a catalyst when only bicarbonate is present (Figure S4e) can slow ozone decomposition almost to half the speed; double the time is required to reach the same residual concentration. This indicates that the catalyst, specifically the synthesized CeTiO_x_, does not facilitate ozone decomposition, allowing more time for the ozone to react in the solution. This can be explained by the ability of the catalyst to provide alternative reaction pathways or by adsorbing ozone molecules, effectively reducing their decomposition rate. As a result, more ozone molecules are retained in the solution, leading to a higher residual concentration. It can be concluded that the presence of a catalyst can alter the kinetics of ozone decomposition and reaction with bicarbonate, leading to a slower overall reaction rate and allowing more ozone to persist in the solution. A similar trend is observed when the bicarbonate is combined with the 2 μM OMP concentration.

The ozone degradation results were explained using the second-order model (Figure 5b). The first-order model was also applied to the data; however, the goodness-of-fit parameter (*R*^2^) was lower than 0.96. In the first-order model, the degradation rate is primarily influenced by changes in ozone concentration. Conversely, the second-order model indicates that factors beyond ozone concentration, such as bicarbonate and OMPs, also impact its degradation rate. In this case, the *R*^2^ was found to be higher, indicating that the model describes the degradation of ozone more precisely. Evaluating the goodness-of-fit parameters for each model helps to determine which one better describes the process and thereby provides insight into the reaction mechanism. The experimental second-order kinetic constants of ozone for the three matrices were as follows: *k*_O3,NaHCO3_ = 135.40 M^−1^ s^−1^, *k*_O3,2μM OMPs_ = 63.38 M^−1^ s^−1^, and *k*_O3,NaHCO3+1g/L CeTiOx_ = 62.72 M^−1^ s^−1^. The constants were determined from the slope of the plot of (1/*C* -1/*Co*) against reaction time. Findings for second-order fitting align with the research of Panda and Mathe [49] as well as Yershov and his collaborators [50].

To better explain the decomposition of ozone in water, the different reactions occurring [50,51,52] are given below. The presence of hydroxyl ions (OH^–^) primarily catalyzes the ozone decomposition, and for the production of a hydroxyl radical (^●^OH), two molecules of O_3_ need to be consumed. The series of reactions leading to ozone decomposition is initiated by the involvement of hydroxyl (^●^OH), hydroperoxyl (HO_2_^●^), and superoxide (O_2_^●–^) radicals. This sequence ends when these radicals recombine, and the chain reaction stops.

(5) $$O_{3}+{OH}^{-}\to {{HO}_{2}}^{-}+O_{2},\;k=70\;M^{-1}s^{-1}$$

(6) $$O_{3}+{{HO}_{2}}^{-}\to {OH}^{•}+{O_{2}}^{•-}+O_{2},\;k=2.8\times 10^{6}\;M^{-1}s^{-1}$$

(7) $$O_{3}+{OH}^{•}\to {{HO}_{2}}^{•}+O_{2},\;k=1.1\times 10^{8}\;M^{-1}s^{-1}$$

(8) $${{HO}_{2}}^{•}↔{O_{2}}^{•-}+H^{+},\;pKa=4.8$$

(9) $$O_{3}+{O_{2}}^{•-}\to {O_{3}}^{•-}+O_{2},\;k=1.6\times 10^{9}\;M^{-1}s^{-1}$$

(10) $${O_{3}}^{•-}+H^{+}\to {{HO}_{3}}^{•},\;k=5.2\times 10^{10}\;M^{-1}s^{-1}$$

(11) $${{HO}_{3}}^{•}\to {OH}^{•}+O_{2},k=1.1\times 10^{5}\;M^{-1}s^{-1}$$

(12) $$O_{3}+{{HO}_{2}}^{•}\to {OH}^{•}+2O_{2},\;k=1.0\times 10^{4}\;M^{-1}s^{-1}$$

(13) $${O_{2}}^{•-}+{{HO}_{2}}^{•}\to {{HO}_{2}}^{-}+O_{2},\;k=1.0\times 10^{9}\;M^{-1}s^{-1}$$

(14) $${{HO}_{2}}^{-}+H^{+}\to {H_{2}O}_{2}$$

In addition to direct decomposition, ^●^OH can be produced through the decomposition of O_3_ on the catalyst surface. The exact mechanism of ^●^OH formation can vary depending on the type of catalyst and reaction conditions; however, the most possible mechanism is the adsorption of ozone onto the catalyst surface, followed by its dissociation into reactive oxygen species. Adsorption occurs through a weak chemical interaction, such as Van der Waals and electrostatic, between the aqueous or gaseous O_3_ molecules and the catalyst’s surface. The catalyst can facilitate the O_3_ decomposition by providing active sites on its surface where the adsorbed water (H_2_O) molecules can form ^●^OH. These reactive species can subsequently react with organic pollutants either adsorbed on the catalyst surface or in the surrounding solution. It is important to note that the catalyst itself does not directly produce ^●^OH. Instead, it acts as a facilitator, providing a favorable environment for O_3_ decomposition to ^●^OH. The following reactions can describe the reaction mechanism when the catalyst (M) is present [53,54,55]:

(15) $$M+H_{2}O\to {M-OH}^{-}+H^{+}$$

(16) $${M-OH}^{-}+O_{3}\to {M-OH}^{•}+{O_{3}}^{•}$$

(17) $${M-OH}^{-}+O_{3}\to {M-{HO}_{2}}^{•}+O_{2}$$

(18) $${M-OH}^{•}+pollutant\to M+product$$

(19) $${M-{HO}_{2}}^{•}+pollutant\to M+product$$

Nevertheless, the reaction mechanism when gaseous O_3_ is applied differs. Many studies have revealed the effectiveness of gaseous ozone in accelerating the generation of free radicals by catalyst [25,39,56]. When O_3_ is applied in wet environments, the presence of water molecules can influence catalyst deactivation during ozone decomposition [17,54]. Studies have shown a significant decrease in MnO_x_ activity under such conditions, with observations of Mn oxidation and structural changes in the catalyst due to water molecules [54,57,58]. A proposed reaction pathway involves water molecules forming surface -OH_2_^+^ groups that interact with ozone, leading to catalyst deactivation. One possible explanation is that the absorbed water molecules resist desorbing and accumulate over time, reducing ozone decomposition rates since active sites are already occupied. However, conflicting views exist regarding the inhibition of ozone molecule adsorption by water molecules. Zhu et al. (2017) proposed that water molecules can form a complex with O_3_ and release HO_3_^●^ that can react and give ^●^OH. Given the practical conditions, encountering humidity in such processes is unavoidable. Consequently, addressing the issue of deactivation in humid environments remains imperative, necessitating further exploration of strategies to enhance the water resistance of the catalysts or to innovate new materials with superior water resistance properties.

### 3.3. Surface Response Methodology for Pharmaceutical Degradation

**Figure 6:**
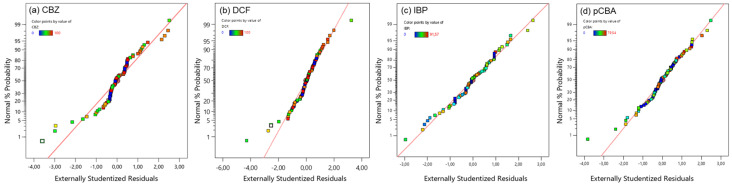
Normal probability plots of residuals of the models used for the removal of (**a**) carbamazepine, (**b**) diclofenac, (**c**) ibuprofen, and (**d**) pCBA under the different catalytic ozonation treatments.

A mixture of micropollutants consisting of three pharmaceuticals (carbamazepine, diclofenac, and ibuprofen) and the pCBA as O_3_/^●^OH probe was used to evaluate the efficiency of the potential catalysts/nanoparticles. The initial concentration of each model compound was 10 μM, and their removal efficiency (% of removal) was plotted in relation to ozone and catalyst dose. A response surface methodology (RSM) was applied to reveal the important variables of their degradation. For the RSM, 80 experiments were performed (five nanoparticles, four ozone doses, and four nanoparticle doses). Before constructing the models, the data were examined to confirm the normality of the externally studentized residuals using the normal probability plot. In Figure 6, it can be observed that residuals from all tested compounds were normally distributed, suggesting the adequacy of the predicted models.

An ANOVA analysis was conducted to assess the models’ validity and adequacy as well as the significant effects and potential interactions among variables. ANOVA is a statistical method used to test hypotheses based on model parameters. The results of the analysis are presented in the Supplementary Material. The factors A, B, and C represent O_3_ dose, NP concentration, and NP type, respectively. There were no interactions between variables such as ozone and nanoparticle dose or type. Furthermore, NP type was not a significant factor in the IBP model, whereas for the other three compounds, the different catalysts only altered the intercept (b_0_) of the equation. The *F*-values for CBZ, DCF, IBP, and pCBA were 1073.35, 807.47, 491.22, and 333.73 respectively. The *p*-values were all <0.0001, an indicator that the polynomial models were highly significant for removing the compounds during catalytic ozonation. According to the results, the factors that influenced the degradation of the four compounds the most were the O_3_ dose and the NP concentration at a lower level.

To depict the mechanism of CBZ, DCF, IBP, and pCBA degradation under heterogeneous catalytic ozonation, polynomial linear regression models were constructed using the degradation outcomes, specifically the percentage (%) of removal in each experimental condition. By examining the coefficients of the resulting equation, formulated in terms of coded factors (x_1_ = A: O_3_ dose; x_2_ = B: NP concentration) through the equations (Equations (20)–(23)), one can determine the relative influence of the factors on the removal of each compound. The statistical analysis and model fitting demonstrated good predictability and accuracy, since the correlation coefficients (*R*^2^) were higher than 0.950.

(20) $${CBZ}_{\%removal}=b_{0}+{1.17x}_{1}-{0.00345x_{1}}^{2},\;R^{2}=0.988$$

(21) $${DCF}_{\%removal}=b_{0}+{1.24x}_{1}-{11.8x}_{2}-{0.00382x_{1}}^{2}-{27.5x_{2}}^{2}-{13.5x_{2}}^{3},\;R^{2}=0.988$$

(22) $${IBP}_{\%removal}=b_{0}+{0.498x}_{1}-{21.6x}_{2}+{50.6x_{2}}^{2}-{26.3x_{2}}^{3},\;R^{2}=0.959$$

(23) $${pCBA}_{\%removal}=b_{0}+{0.541x}_{1}-{10.0x}_{2}-{0.000604x_{1}}^{2}+{27.4x_{2}}^{2}-{14.9x_{2}}^{3},\;R^{2}=0.970$$

Table 4 illustrates the different intercept values (b_0_) for the models affected by the NP type. The results of the correlation analysis suggest that OMPs degradation is mainly explained by the O_3_ dose with CBZ and DCF being the most affected (higher coefficients). CBZ was the only compound that NP concentration (x_2_) did not have a significant role in its degradation; however, the other compounds were negatively affected by that factor.

**Figure 7:**
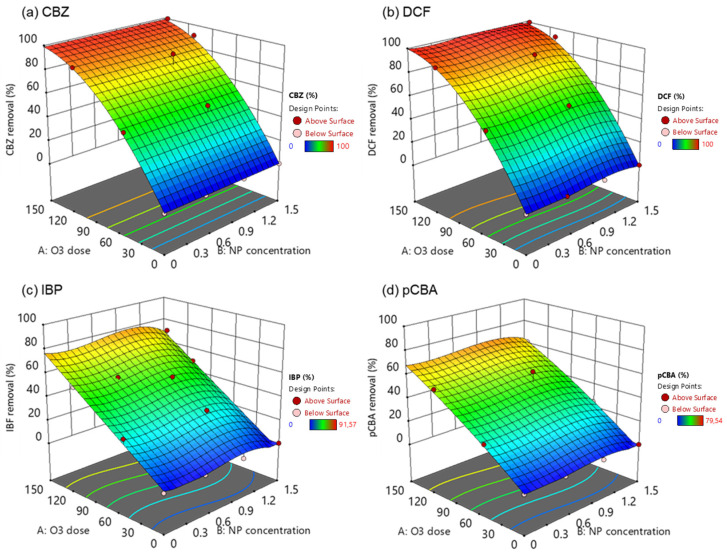
Response surface plots in 3D for the removal of (**a**) carbamazepine, (**b**) diclofenac, (**c**) ibuprofen, and (**d**) pCBA under the different catalytic ozonation treatments.

The effects of the independent variables (O_3_ dose and NP concentration) on the response variable, i.e., the removal of pharmaceuticals and pCBA, were presented in three-dimensional (3D) surface plots (Figure 7). In these experiments, the ozone dose was added at the beginning of the experiment. It was left to react for 4 h before measuring the residual concentration of the OMPs, and the time allowed for the complete depletion of ozone and the inactivation of the radical chain pathway. Even though contact time plays a crucial role in the effective and efficient removal of OMPs, especially in wastewater treatment, the study tried to elucidate the catalyst’s behavior under low and high ozone doses. It could not predict the optimum NP concentration for the effective degradation of each compound, and it was obvious that the higher the O_3_ dose, the higher the removal.

CBZ and DCF are compounds that can quickly degrade by ozone (k_O3/CBZ_ = 3.00 × 10^5^ M^−1^ s^−1^, k_O3/DCF_ = 6.85 × 10^5^ M^−1^ s^−1^) because they contain functional groups that are susceptible to attack by ozone, such as double bonds, aromatic rings, and heteroatoms (e.g., nitrogen and oxygen). This is the main reason behind the complete elimination of those two compounds in high ozone doses (150 μM). The addition of the nanoparticle in the oxidation process has not increased the degradation rates, and these findings were also supported by Lara-Ramos et al. [59] and Fan et al. [60]. Their studies found that ozone dose was the most significant effective parameter for DCF and thymol degradation, respectively. Interestingly, increasing the catalyst load was found to reduce the degradation percentages of DCF.

On the other hand, IBP and pCBA are molecules recalcitrant to ozone oxidation due to their low reactivity with ozone (*k*_O3/IBP_ = 9.6 M^−1^ s^−1^, *k*_O3/pCBA_ < 0.15 M^−1^ s^−1^). Still, they react with hydroxyl radicals, and their incomplete degradation in high ozone doses implies the inability of the system to produce high ^●^OH concentrations. From the surface plots, it can be concluded that when the catalyst concentration was at 1.5 g L^−1^, the removal was lower than the 1.0 g L^−1^. This finding suggests that an excess amount of catalyst can hinder the ozone decomposition to ^●^OH. Based on previous studies, catalysts’ performance was negatively affected under humid environments [54]. Active sites on the catalyst surface could be saturated with water molecules. Another explanation is that the available O_3_ immediately reacted with CBZ and DCF, thus reducing its concentration and, subsequently, ^●^OH generation.

The application of RSM in this study enabled a thorough evaluation of the influence of multiple variables on the degradation of pharmaceuticals during heterogeneous catalytic ozonation. RSM identified the transferred ozone dose (TOD) as the most significant factor, emphasizing the critical role of ozone availability in driving pharmaceutical degradation. Remarkably, RSM demonstrated that, under the tested conditions, the effect of TOD outweighed any catalytic enhancement provided by the nanoparticles, reinforcing the importance of ozone concentration. This method offered technical advantages by simplifying the experimental design and reducing the number of experiments required while providing statistically robust results. Through RSM, optimal operating conditions were identified, and the interaction effects between ozone concentration and nanoparticle dosage were clarified. Ultimately, RSM proved to be an efficient tool for process optimization, allowing a deeper understanding of the catalytic ozonation process and the limited role of the catalysts in the studied system.

### 3.4. Degradation of Pharmaceuticals

**Figure 8:**
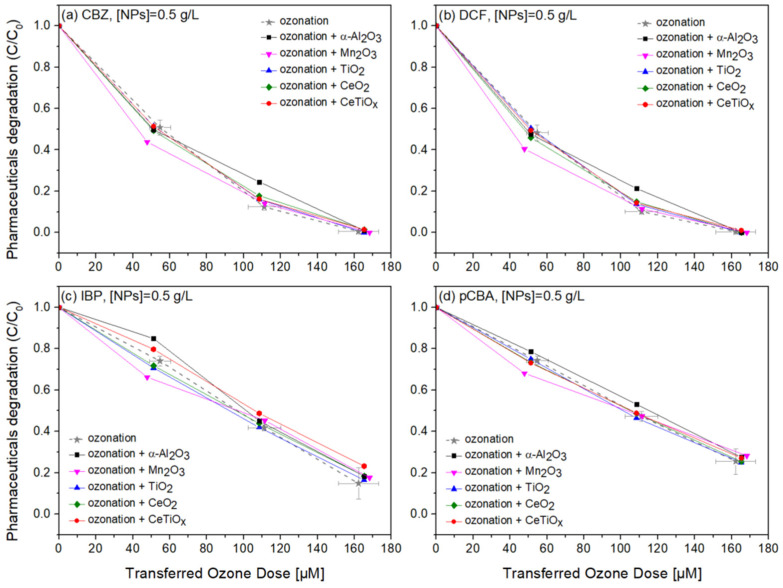
Degradation graphs of (**a**) carbamazepine, (**b**) diclofenac, (**c**) ibuprofen, and (**d**) pCBA treated with different ozone doses (0–150 μM) with nanoparticles concentration at 0.5 g L^–^**^1^**.

**Figure 9:**
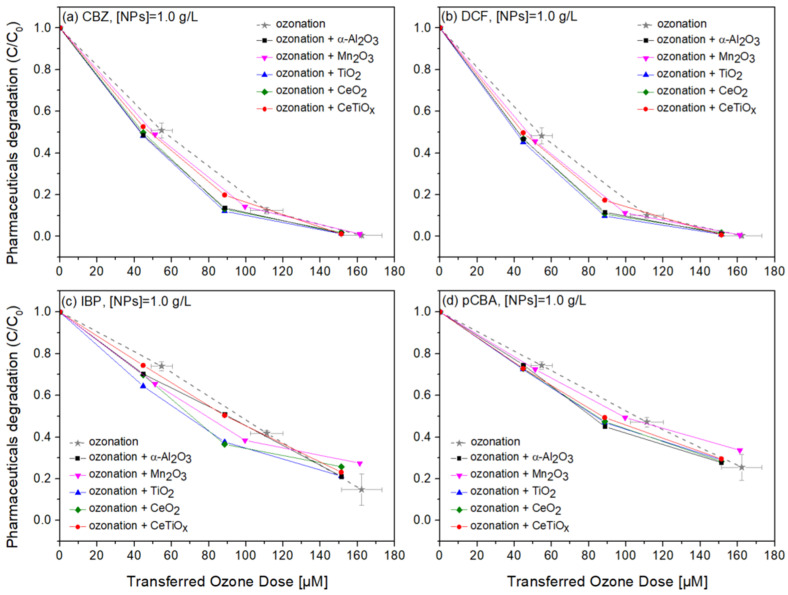
Degradation graphs of (**a**) carbamazepine, (**b**) diclofenac, (**c**) ibuprofen, and (**d**) pCBA treated with different ozone doses (0–150 μM) with nanoparticles concentration at 1.0 g L^−1^.

**Figure 10:**
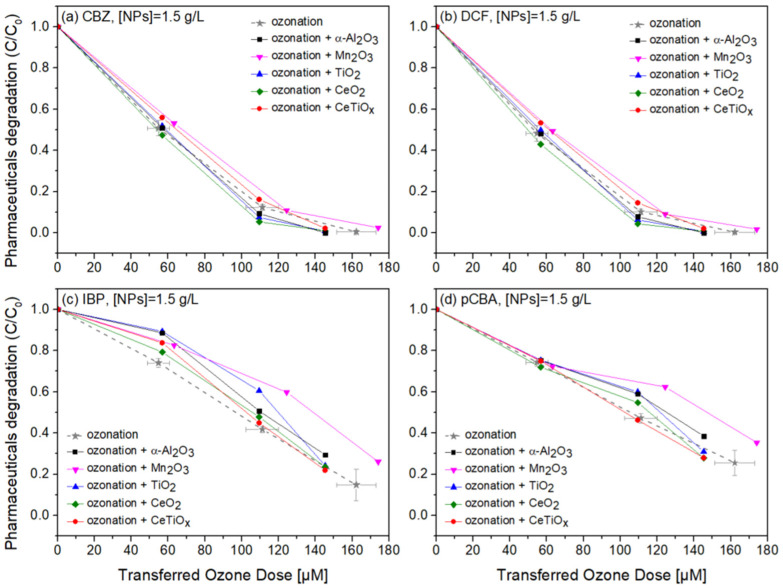
Degradation graphs of (**a**) carbamazepine, (**b**) diclofenac, (**c**) ibuprofen, and (**d**) pCBA treated with different ozone doses (0–150 μM) with nanoparticles concentration at 1.5 g L^−1^.

Carbamazepine, diclofenac, and ibuprofen are considered recalcitrant compounds because they resist degradation in natural environments. Ozone treatment effectively breaks down these persistent compounds into less harmful by-products, facilitating their removal from water or wastewater [61,62,63]. Therefore, the degradation of those pharmaceuticals in the presence of pCBA, an ^●^OH scavenger, in a heterogeneous catalytic system was studied. Figure 8, Figure 9 and Figure 10 display the normalized degradation of the tested OMPs in three NPs concentrations, 0.5, 1.0, and 1.5 g L^−1^, respectively. Theoretical ozone doses varied from 0–150 μM. However, the experimental concentrations deviated; standard errors for the x and z axes are noted with a grey color on the ozonation alone treatment.

Four commercially available nanoparticles, α-Al_2_O_3_, Mn_2_O_3_, TiO_2_, and CeO_2_, were employed to investigate their potential catalytic effect on the degradation of selected pharmaceuticals during the ozonation process. Additionally, we synthesized Ce-doped TiO_2_ (CeTiO_x_) using the sol–gel method at a 1% molar ratio of Ce to Ti. The synthesized CeTiO_x_ was included based on previous studies, such as Lee et al. (2021) [33], which demonstrated its promising performance in advanced oxidation processes (AOPs). Specifically, in batch ozonation experiments with higher ozone doses (5 mg L^−1^), CeTiO_x_ significantly enhanced the degradation of DEET, with negligible adsorption effects, suggesting its potential to improve catalytic ozonation.

Notably, in Figure 8, CBZ and DCF data follow similar trends due to their affinity towards ozonation. At the same time, IBP and pCBA demonstrate a different trend with lower degradation rates. It is apparent that the higher the ozone dose, the higher the degradation for all compounds independently of the nanoparticle used. There is evidence that the presence of α-Al_2_O_3_, Mn_2_O_3_, TiO_2_, CeO_2_, and CeTiO_x_ did not accelerate the removal of the compounds, especially the ones that do not directly react with ozone (IBP and pCBA). The interaction plots (Figure S5) derived from the RSM analysis also support that there is no effect present, and the response mean (% of removal) is the same across all factor levels (NP type). These results reflect those of Pocostales et al. [64] who also found that among other pharmaceuticals, DCF was quickly removed by ozonation and the presence of the commercial γ-Al_2_O_3_ or the synthesized Co_3_O_4_/Al_2_O_3_. However, these catalysts impacted the mineralization of the pharmaceutical compounds used. For evaluating the performance of catalytic ozonation on each pharmaceutical compound, it would be better to test each separately. Nevertheless, this situation would not represent the actual conditions when a mixture of compounds is present in wastewater.

From the adsorption experiments, the mixture of micropollutants was left for equilibrium with NPs for one hour before the addition of ozone. No significant difference was found in all micropollutant concentrations. Less than 1% of the compounds were adsorbed on the surface of the catalysts, suggesting that there was not an actual collision phenomenon occurring between the material and the organic molecules. Similar findings were reported by Lee and his collaborators when using their synthesized CeTiO_x_ material to degrade DEET. This could be again explained by the adsorption competition phenomena occurring on the catalyst’s active sites. Chen and colleagues [65] showed that the catalyst’s active sites were consistently taken up by water molecules that were unable to be desorbed, consequently diminishing its catalytic activity. Likewise, Liu et al. [17] found that water vapor generated during the catalytic ozonation process progressively accumulated on the catalyst surface, thus affecting the ability of the catalyst to further degrade the formaldehyde.

To further evaluate the effect of the NPs, individual graphs of each compound with different concentrations of the catalysts were generated (Figure S6a). Comparing the different treatments regarding OMPs’ removal was visually easier from those data. It can be seen that Mn_2_O_3_ has a low performance at the highest dose, 1.5 g L^−1^. The catalyst dose giving slightly better degradation at 100 μM TOD was 1.0 g L^−1^ for almost all NPs. However, no substantial difference was observed. Interestingly, in Figure 10c,d, ozonation itself gave better degradation results than when the NPs were present. The higher catalyst dose (1.5 g L^−1^) declined the degradation efficiency of the compounds, and this can be attributed to the increased turbidity of the solution [66].

Optimizing the concentration of catalysts in heterogeneous catalytic ozonation proved essential, with 1 g L^−1^ offering efficient pharmaceutical degradation at reduced costs by minimizing turbidity and active site saturation. The enhanced degradation of compounds like CBZ and DCF highlights the process’s suitability for pollutants with high ozone reactivity. Moreover, our findings point to the potential of heterogeneous catalytic ozonation to provide additional benefits beyond micropollutant removal, including a reduction in membrane fouling and possible flux improvements when applied in hybrid systems. Future work should explore catalyst reusability and integration with continuous-flow systems to maximize process’s practical application in large-scale treatment.

It is also well-documented that the combination of noble metal oxides is more effective when ozonation is applied as they offer oxygen vacancies for adsorption or/and promote the dissociation of intermediate species to further mineralize OMPs [14,16,17]. It was expected that the synthesized nanoparticle, CeTiO_x_, would exhibit high catalytic activity due to its attractive characteristics, such as mesoporous structure, negative charge, and the coexistence of the redox couples Ti^3+^/Ti^4+^ and Ce^3+^/Ce^4+^ [16,33]. Exhibiting good performance when used for DEET degradation [33], it was believed that it would perform the same in the degradation of IBP and pCBA. It is important, though, to understand in what kind of environments the catalyst is applied. When gaseous O_3_ is applied, it is more likely to favor the O_3_ molecule adsorption, facilitating more reaction pathways, such as the generation of more ^●^OH (Equations (6), (10)–(12)).

### 3.5. Catalyst-Pharmaceutical Interactions: Challenges and Future Directions

Undeniably, the catalysts’ characteristics are crucial in catalytic ozonation [67,68]. The efficiency of ozone activation and subsequent targeted pollutant degradation is closely related to the nanoparticles’ physicochemical properties. The specific surface area, pore size distribution, and zeta potential significantly determine the catalytic performance. In this study, α-Al_2_O_3_, with its high specific surface area and pore volume, offers many active sites for catalysis. However, its macroporous structure limits its interaction with smaller molecules like pharmaceuticals, reducing its effectiveness in generating hydroxyl radicals. In contrast, CeTiO_x_, with a predominantly mesoporous structure (pores < 10 nm), facilitates better mass transfer and more efficient interactions between ozone and pollutants. In addition, the H2(a) hysteresis loop in CeTiO_x_ indicates the presence of bottlenecks in its pores, which enhances the trapping and reaction of ozone, improving pollutant degradation efficiency.

Additionally, the XRD analysis confirmed that CeTiO_x_ retained the anatase phase of TiO_2_, known for its high catalytic activity, which, combined with favorable pore characteristics, enhances its performance in catalytic ozonation. Zeta potential measurements also support this, as surface charge influences the interaction between nanoparticles and ozone. In this study, NaHCO_3_ was used to maintain a pH of 7.6–7.8, replicating typical conditions in a wastewater treatment plant. Under these conditions, the catalysts exhibited distinct surface charges due to their varying points of zero charge (PZC). Specifically, Al_2_O_3_ (PZC = 8.6) was positively charged, Mn_2_O_3_ (PZC = 7.2) was slightly positive, CeO_2_ (PZC = 6.9) and CeTiO_x_ (PZC = 3.7) were negatively charged, and TiO_2_ (PZC = 7.7) remained neutral. The different charge states play a role in their interactions with both ozone and pharmaceutical compounds. The four model OMPs used also present various charges and behaviors under the study’s pH conditions, with CBZ being protonated (*pKa* > pH) and the other three compounds (DCF, IBU, and pCBA) deprotonated (*pKa* < pH). This charge variation affects each compound’s electron-donating or -accepting tendencies, influencing their interactions with charged catalysts during ozonation. Deprotonated compounds, being electron-rich, are likely to act as electron donors, while the protonated carbamazepine, electron-poor in this pH, acts as an electron acceptor. Furthermore, studies like those of Lee et al. [33] and Ćurković et al. [34] have shown that CeTiOx, with a 1% mol Ce to Ti ratio, improves degradation efficiency compared to other ratios. Overall, CeTiO_x_’s mesoporous structure and stable crystal form make it a strong candidate for enhancing catalytic ozonation, especially when targeting micropollutants like pharmaceuticals.

However, while CeTiO_x_ showed promising structural and surface characteristics, its performance in catalytic ozonation was not significantly better than other nanoparticles, likely due to the challenges posed by using ozone in the liquid phase. In catalytic ozonation, effective ozone activation requires sustained interaction between ozone and the catalyst surface to generate reactive oxygen species like hydroxyl radicals. The liquid-phase ozone may have limited contact time with the catalyst’s active sites, especially when nanoparticles like CeTiO_x_ are involved, leading to minimal ozone activation efficiency. This could explain why the expected enhancement in degradation efficiency was not observed in this study. Furthermore, while CeTiO_x_’s mesoporous structure and moderate specific surface area should theoretically promote better ozone interaction and degradation, the rapid depletion of ozone and the liquid–solid interface limitations hindered this potential. In several cases, ozonation alone outperformed catalytic ozonation, which suggests that the catalysts were not effectively decomposing ozone into hydroxyl radicals as hypothesized.

It is important to note that the metal nanoparticle size distributions, as reported in Table 3, did not show a direct correlation with the degradation of pharmaceuticals in Figure 10. Despite the smaller particle sizes of Mn_2_O_3_ and CeO_2_ (28 nm), their performance was not significantly better than α-Al_2_O_3_, which had a much larger particle size of 78 nm. This suggests that other factors, such as surface chemistry, the ability to adsorb and activate ozone molecules, and pore structure, play a more critical role in catalytic performance. In aqueous ozone systems, the limited contact time and the potential saturation of active sites with water molecules may also reduce the influence of particle size on degradation. Therefore, while nanoparticle size is an important parameter, the overall physicochemical properties of the nanoparticles must be considered when evaluating their catalytic efficiency in pharmaceutical removal.

Building on these findings, it becomes evident that while CeTiO_x_ and other nanoparticles exhibit promising physical characteristics, their efficiency in activating ozone and enhancing pollutant degradation in a liquid phase remains limited. The key challenge lies in the insufficient interaction between the liquid-phase ozone and the catalyst surface, reducing the formation of reactive oxygen species necessary for effective degradation. In some instances, ozonation alone provided better degradation results than catalytic ozonation, underscoring the need to revisit the application of heterogeneous catalysts under these conditions.

Therefore, future studies should focus on optimizing the interaction between catalysts and ozone, either by modifying the catalyst preparation process or by exploring alternative operational strategies. Subsequent experiments can focus on longer reaction times and the use of continuous flow systems where ozone is introduced consistently, allowing for sustained contact with the catalysts in a different type of reactor. Additionally, the ozone flow rate and the method of ozone introduction (gaseous or dissolved) can be optimized to maximize the interaction between ozone and the catalyst surface. In this context, employing gaseous ozone might extend the interaction time by preventing the rapid dissolution and subsequent saturation of ozone in the aqueous phase

In addition to exploring gaseous ozone, optimizing the catalyst structure and surface properties to reduce water adsorption could be another strategy to overcome this inhibition. For example, hydrophobic modifications to the catalyst surface by calcination before use could minimize water saturation and allow for more efficient ozone adsorption and radical generation. This approach has been investigated in other catalytic processes [69] and could be adapted for heterogeneous catalytic ozonation. Moreover, one potential approach could be to optimize the synthesis of catalysts with improved redox properties, such as incorporating transition metals or mixed metal oxides [70,71] that promote higher radical production. Additionally, advanced catalyst modifications, including surface functionalization or doping with heteroatoms, may increase the selectivity towards hydroxyl radical formation, thus broadening the scope of micropollutants that can be effectively degraded.

Finally, there was an attempt to calculate the *Rct* values, i.e., the exposure of the compounds to both O_3_ and ^●^OH [19]. However, the high organic micropollutant (OMP) load caused ozone to be consumed too quickly, preventing a reasonable estimation of the generated ^●^OH. To better evaluate the effect of catalytic ozonation on the degradation of pharmaceuticals or other OMPs, it is recommended to apply ozone in its gaseous form and focus on one compound at a time. This approach would allow the experiment to concentrate on the mechanistic pathways of degradation and the activation of the catalyst, potentially offering more precise insights into the catalyst’s performance and the efficiency of the overall process, especially when working with secondary effluents from urban wastewater, where the matrix is far more complex. Understanding the characteristics of the matrix can also help identify the optimum conditions for degradation, which are the most harmful ones, effectively and efficiently.

## 4. Conclusions

The main conclusions derived from this study are:

- CeTiO_x_ was successfully synthesized using the sol–gel method. This catalyst, prepared with a 1% molar ratio of Ce/Ti, exhibited promising structural properties, though its catalytic efficacy in this study was limited.
- Response surface methodology (RSM) proved to be an effective tool for optimizing the removal of pharmaceuticals by identifying the most significant variables influencing the ozonation process. The transferred ozone dose (TOD) was the most impactful among the tested variables.
- Ozone decomposition kinetics were found to vary significantly based on organic load. In low organic load conditions (with bicarbonate present), the decomposition of aqueous ozone followed second-order kinetics. However, under high organic load, ozone was consumed too rapidly for second-order kinetics to apply, pointing to instantaneous ozone demand.
- The short contact time of aqueous ozone with the nanoparticles (NPs) resulted in limited catalytic activity. In this study, catalytic ozonation did not provide a significant advantage over ozonation alone, mainly due to the rapid depletion of aqueous ozone in the matrix. The catalytic effect was negligible because ozone had insufficient time to interact with the catalyst surface, with only around 20% of the ozone dose being converted into hydroxyl radicals (^●^OH).
- The rapid depletion of aqueous ozone in the matrix rendered *Rct* calculations infeasible. These findings suggest that catalytic ozonation may not be necessary or effective in systems where ozone reacts quickly with the matrix and that direct ozonation may suffice in such cases. However, catalytic ozonation could benefit systems where ozone persists longer, allowing for more substantial interaction with the catalyst.
- The efficacy of catalytic ozonation can be hindered by aqueous ozone due to the saturation of catalyst surface sites with water molecules. Gaseous ozone applications, in contrast, may enhance catalytic efficiency by promoting better ozone–catalyst interaction.
- While batch processes are valuable for preliminary studies and offer controlled environments for investigating catalyst behavior, they do not always replicate the conditions of large-scale, continuous-flow systems typically used in wastewater treatment plants
- Finally, a detailed understanding of the ozone-catalyst interaction mechanism in heterogeneous catalysis is essential for optimizing catalytic processes. This knowledge enables selecting and designing more effective catalysts, particularly in systems where maximizing ozone conversion into hydroxyl radicals is crucial for degradation.

## Figures and Tables

**Table 1:** Physiochemical characteristics of model organic micropollutants.

Micropollutant	Molecular Structure	Molar Mass [g mol^−1^] *	Log Kow *	pKa *
Carbamazepine (CBZ)	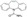	236.27	2.45	13.9
Diclofenac (DCF)	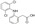	296.15	4.51	4.15
Ibuprofen (IBP)	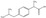	206.29	3.97	5.3/4.4
para-Chlorobenzoic acid (pCBA)	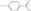	156.57	2.65	3.98

* data received from the National Library of Medicine USA, https://pubchem.ncbi.nlm.nih.gov/ (accessed on 22 January 2020).

**Table 2:** Selected factors and response variable.

Factors	Class	Levels				
x_1_: Transferred Ozone dose [μM]	Numerical	0	50	100	150	
x_2_: Nanoparticles concentration [g L^−1^]	Numerical	0	0.5	1	1.5	
x_3_: Nanoparticles type	Categorical	α-Al_2_O_3_	CeO_2_	Mn_2_O_3_	TiO_2_	CeTiO_x_
y: % removal for CBZ, DCF, IBP, and pCBA	Response					

**Table 3:** Data from BET analysis for each tested nanoparticle.

Parameter	Symbol, Unit	α-Al_2_O_3_	Mn_2_O_3_	TiO_2_	CeO_2_	CeTiO_x_
Specific surface	*S_BET_*, m^2^ g^−1^	147.8	18.6	49	41.3	36.4
Pore volume	*V_pores_*, cm^3^ g^−1^	0.902	0.059	0.169	0.157	0.043
Micropore Volume (<2 nm)	*V_micropores_*, cm^3^ g^−1^	0.002	0.0007	-	0.0005	-
Mean pore diameter	*d*_pore_, nm	22.9	11.2	8.3	14.1	4.5
Mean particle size	*d_patricle_*, nm	78	28	28	28	-

**Table 4:** Intercept values (b_0_) for the models affected by the type of nanoparticles used in catalytic ozonation experiments.

Model Intercept	Nanoparticles Type				
b_0_	α-Al_2_O_3_	Mn_2_O_3_	CeO_2_	TiO_2_	CeTiO_x_
CBZ	0.345	−1.95	1.43	1.30	−1.51
DCF	0.327	−1.62	1.58	1.06	−1.52
IBP *	1.79	1.79	1.79	1.79	1.79
pCBA	0.314	−2.22	1.97	1.38	1.004

* All offset values are identical for IBP because no significant influence for the catalyst was evidenced in the statistical analysis.

## Data Availability

All data are accessible upon request to the corresponding author.

## Supplementary Materials

The following supporting information can be downloaded at: https://www.mdpi.com/article/10.3390/nano14211747/s1, Figure S1. Scheme for the preparation of the CeTiO_x_ nanoparticles; Table S1. Technical properties of the commercially available nanoparticles; Figure S2. Ozone-treated indigo solutions with the addition of different volumes (on the left) and the ozone calibration curve using the indigo method (on the right); Table S2. HPLC-UV mobile phase; Table S3. Retention times, wavelengths, LoQ, and LoD for each tested compound; Figure S3. Calibration curves for the model compounds (a) amoxicillin, (b) carbamazepine, (c) *para*-chlorobenzoic acid, (d) diclofenac sodium, and (e) ibuprofen.; Figure S4. Degradation of ozone over time (in seconds) in different matrices for exploring the effects of OMPs’ concentration, bicarbonate, and catalyst presence (TOD = 100 μM, 240 mL treated volume, [NaHCO_3_] = 1 mM, [OMPs] = 2 or 10 μM, catalyst concentration = 1 g^−1^); Figure S5. Interaction plots of the tested variables (NPs concentration, NPs type, and ozone dose) for CBZ, DCF, and pCBA; Table S4. ANOVA analysis; Figure S6a. Degradation of CBZ, DCF, IBP, and pCBA (starting from left to right) under three different ozone doses (50–150 μΜ) in the presence of metal oxides; α-Al_2_O_3_, Mn_2_O_3_, TiO_2_, CeO_2_, and CeTiO_x_ (starting from top to bottom) at different concentrations; 0.5 (green), 1.0 (blue), and 1.5 (red) g L^−1^.; Figure S6b. Degradation of CBZ (green), DCF (blue), IBP (orange), and pCBA (red) under different ozone doses (0–150 μM).
