# Supplementary material for: Heterogeneous Catalytic Ozonation of Pharmaceuticals: Optimization of the Process by Response Surface Methodology
Source: Nanomaterials (Basel). 2024 Oct 30;14(21):1747. doi: 10.3390/nano14211747 (PMC11547524; doi:10.3390/nano14211747)
Supplement: Supplementary file 1 [file nanomaterials-14-01747-s001.zip › nanomaterials-3238646-supplementary.pdf]

Supplementary material

Heterogeneous Catalytic Ozonation of pharmaceuticals: Optimization of the process by Response Surface Methodology

Nikoletta Tsiarta [1,2,4], Wolfgang Gernjak [1,3,*], Hrvoje Cajner [4], Gordana Matijašić[5], Lidija Ćurković [4,*]

[1] Catalan Institute of Water Research, Carrer Emili Grahit 101, 17003 Girona, Spain; ntsiarta@icra.cat
[2] University of Girona, Campus de Montilivi, 17003 Girona, Spain
[3] Catalan Institution for Research and Advanced Studies (ICREA), 08010, Barcelona, Spain; wgernjak@icra.cat
[4] Faculty of Mechanical Engineering and Naval Architecture, University of Zagreb, Ivana Lučića 5, 10002 Zagreb, Croatia; hrvoje.cajner@fsb.hr, lidija.curkocic@fsb.hr
[5]Faculty of Chemical Engineering and Technology, University of Zagreb, 10000 Zagreb, Croatia; gmatijas@fkit.hr
[*] Correspondence: wgernjak@icra.cat, lidija.curkocic@fsb.hr

1. Scheme of CeTiOx synthesis

The Ce-doped Ti particles (Ce 1% mol with regards to Ti) were synthesized using the sol-gel method, as shown below.

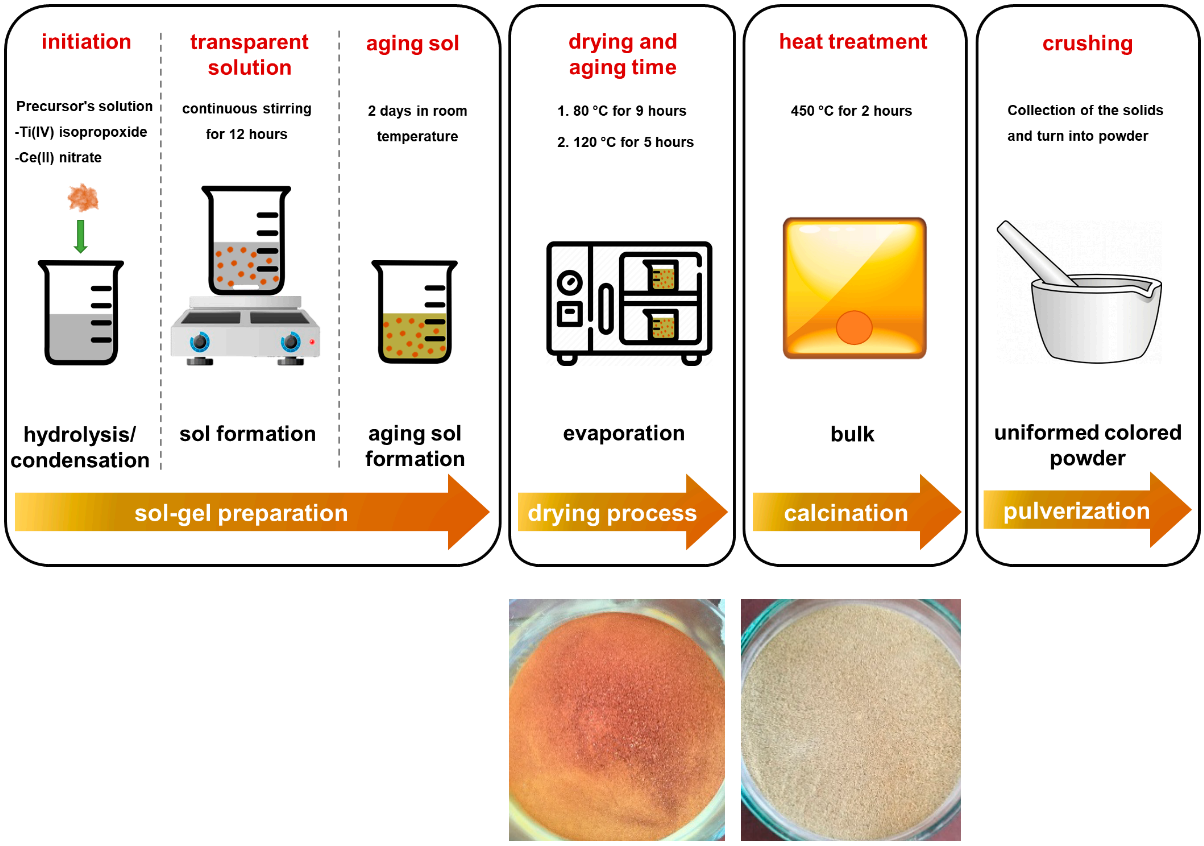

Figure S1. Scheme for the preparation of the CeTiOx nanoparticles.

2. Characterization analysis of the commercially available metal-oxides

In this study, four commercially available metal-oxides/nanoparticles were used: Al_2O_3, CeO_2, Mn_2O_3 and TiO_2 (P25). The data provided in the table below (Table S1) were taken from the manufacturer (Nanografi Nanotechnology, Ankara, Turkey).

Table S1. Technical properties of the commercially available nanoparticles

	**Commercially available metal-oxides**			
**Technical properties**	**α-Al_2O_3**	**CeO_2**	**Mn_2O_3**	**TiO_2**
Average diameter [nm]	78	8.0-28.0	28	34
purity [%]	99.5	99.975	99.4	99.9
colour	white	light yellow	brown	white
Morphology	nearly spherical	N/A	N/A	N/A
Crystallographic Structure	rhombohedral	cubic crystal	cubic bixbyite structure	Tetragonal (Ditetragonal dipyramidal)
Specific Surface Area [m^2g^{-1}]	>20	35-55	>155	54
True density [g cm^{-3}]	3.9	7.2	4.5	4.3
Elemental Analysis	Ca <20 ppm<br>V <5 ppm<br>Cl <280 ppm<br>Co <5 ppm<br>Na <30 ppm<br>Mn <5 ppm	CaO ≤0.005<br>Fe_2O_3 ≤0.002<br>MgO ≤0.001<br>La_2O_3 ≤0.008<br>TiO_2 ≤0.001	N/A	N/A

3. Indigo method for the quantification of ozone concentration

The indigo solution was prepared based on Bader & Hoigne (1981). This method is very sensitive, precise, fast, specific, and easy to handle. It was developed for analyzing aqueous ozone and it was adjusted to the matrix of the experiment. The ozone concentration of the saturated ozone solution prepared in demineralized water was determined using a UV-Vis spectrophotometer (Shimadzu UV-1800, Shimadzu Corporation, Japan) at a wavelength of 260 nm using a quartz cuvette with 1 cm path length. The molar adsorption coefficient of ozone (ε_{260} = 3200 cm^{-1} M^{-1}) and the Beer-Labert law were utilized to estimate the concentration of the saturated solution.

Using a glass pipette, specific volumes of the saturated solution were transferred to the reactor bottles to achieve the theoretical transferred ozone dose (TOD), i.e., 50-, 100-, and 150 μM of TOD. Subsequently, a 2 or 4 mL aliquot of the ozone solution was transferred into a 50 mL volumetric flask containing 5 mL of phosphate buffer at pH = 2 (85% H_3PO_4 and NaH_2PO_4) and 1 mL of 1 mM indigo solution (dissolved in 20 mM H_3PO_4). The flasks were then filled with demineralized water, and the absorbance of the solutions was measured at 600 nm. For the extraction of the calibration curve, specific volumes of the saturated ozone solution, ranging from 150 – 4000 μL, were added to various volumetric flasks (50 mL) containing the buffer and the indigo solution. The different solutions were measured at 600 nm, and a calibration curve of absorbance Vs ozone molar concentration was constructed with a maximum quantification limit of 12.6 μM (0.6 mg L^{-1}) of O_3. The calculated molar coefficient at 600 nm (ε_{600} = 25115 cm^{-1} M^{-1}) was finally used to calculate the initial ozone concentration in each reactor bottle.

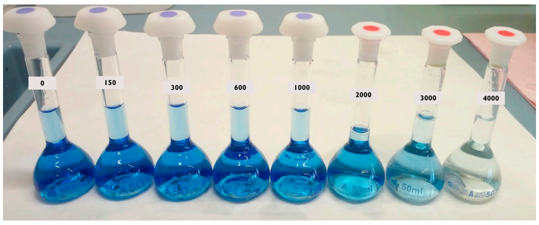

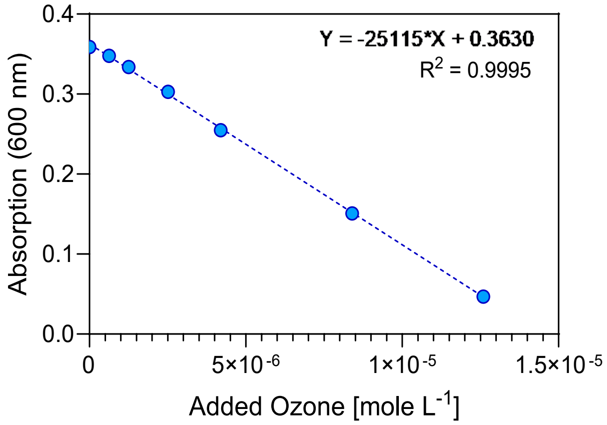

Figure S2. Ozone-treated indigo solutions with the addition of different volumes (on the left) and the ozone calibration curve using the indigo method (on the right).

4. HPLC method for the qualification and quantification of the model compounds

For the quantification and qualification of the model compounds a method was developed in the HPLC-UV Agilent 1200 (Agilent Technologies, Santa Clara, CA, USA). The instrument was equipped with a quaternary pump, an autosampler, and an ultraviolet-visible detector. A C18 column (Microsorb-MV 100-5 250 × 4.6 mm) at a working temperature of 30 °C and flow 0.8 ml min^{-1} was used to separate the compounds. The method time was set to 40 minutes with an injection volume of 200 μL, and it can successfully separate and quantify the following compounds: amoxicillin, carbamazepine, pCBA, diclofenac, and ibuprofen. For the purpose of the experiments, amoxicillin was decided not to be used.

Three different solvents were used: (A) milli-Q water (H_2O), (B) 100% Acetonitrile (ACN), and (C) a solution containing 0.3% formic acid and 10% ACN. The selected OMPs were separated by changing the percentage of aqueous (H_2O) or organic (ACN) solvent while keeping the acidic water (pH 3) always at 10%. More hydrophilic compounds were analyzed with a higher H_2O:ACN mobile phase mixture, whereas more hydrophobic compounds with a lower H_2O:ACN ratio to avoid elution peaks overlapping. The HPLC gradient of the mobile phase used is given below (Table S2):

Table S2. HPLC-UV mobile phase

Time (min)	A (%)	B (%)	C (%)
0	80	10	10
4	80	10	10
5	80	10	10
8	40	50	10
9	40	50	10
27	40	50	10
28	0	70	30
36	0	70	30
38	80	10	10
40	80	10	10

Five different detector wavelengths were set to identify the OMPs. The retention times and the wavelengths are given in Table S3b. The peaks were quantified by calculating the area under the elution peak at the corresponding detector wavelength, and the concentration of each compound was then obtained from a calibration curve (Figure S3).

Table S3. Retention times, wavelengths, LoQ, and LoD for each tested compound

Organic micropollutant	Retention time (min)	Wavelength	LoQ	LoD
Amoxicillin (AMX)	7.4	228 nm	0.145	0.043
Carbamazepine (CBZ)	16.3	284 nm	0.104	0.031
para-chlorobenzoic acid (pCBA)	17.4	236 nm	0.141	0.042
Diclofenac (DCF)	27.6	274 nm	0.192	0.057
Ibuprofen (IBP)	29.2	222 nm	0.521	0.156

The calibration curves for each compound are given below.

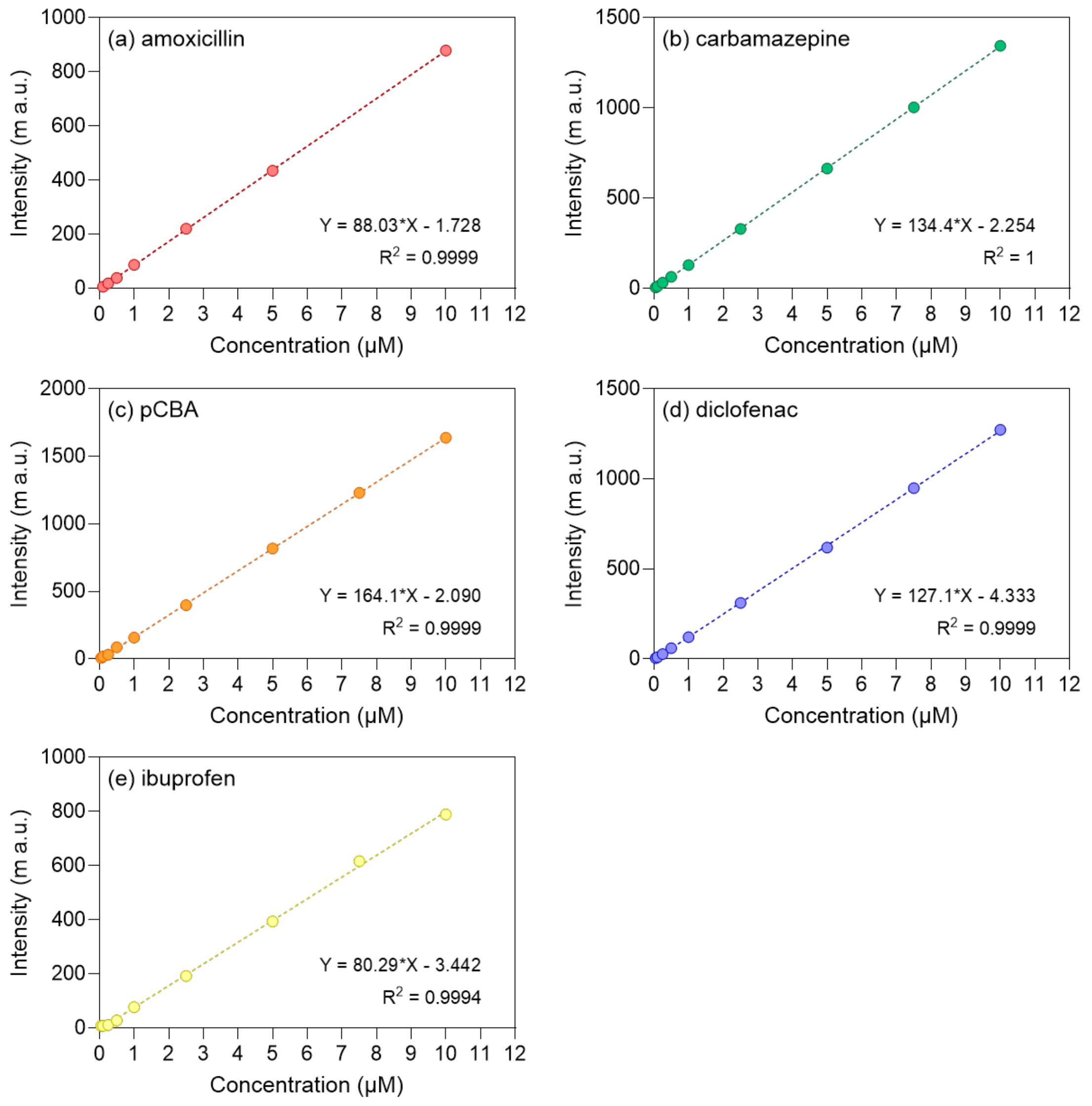

Figure S3. Calibration curves for the model compounds (**a**) amoxicillin, (**b**) carbamazepine, (**c**) *para*-chlorobenzoic acid, (**d**) diclofenac sodium, and (**e**) ibuprofen.

5. Effect of different matrices on ozone decomposition

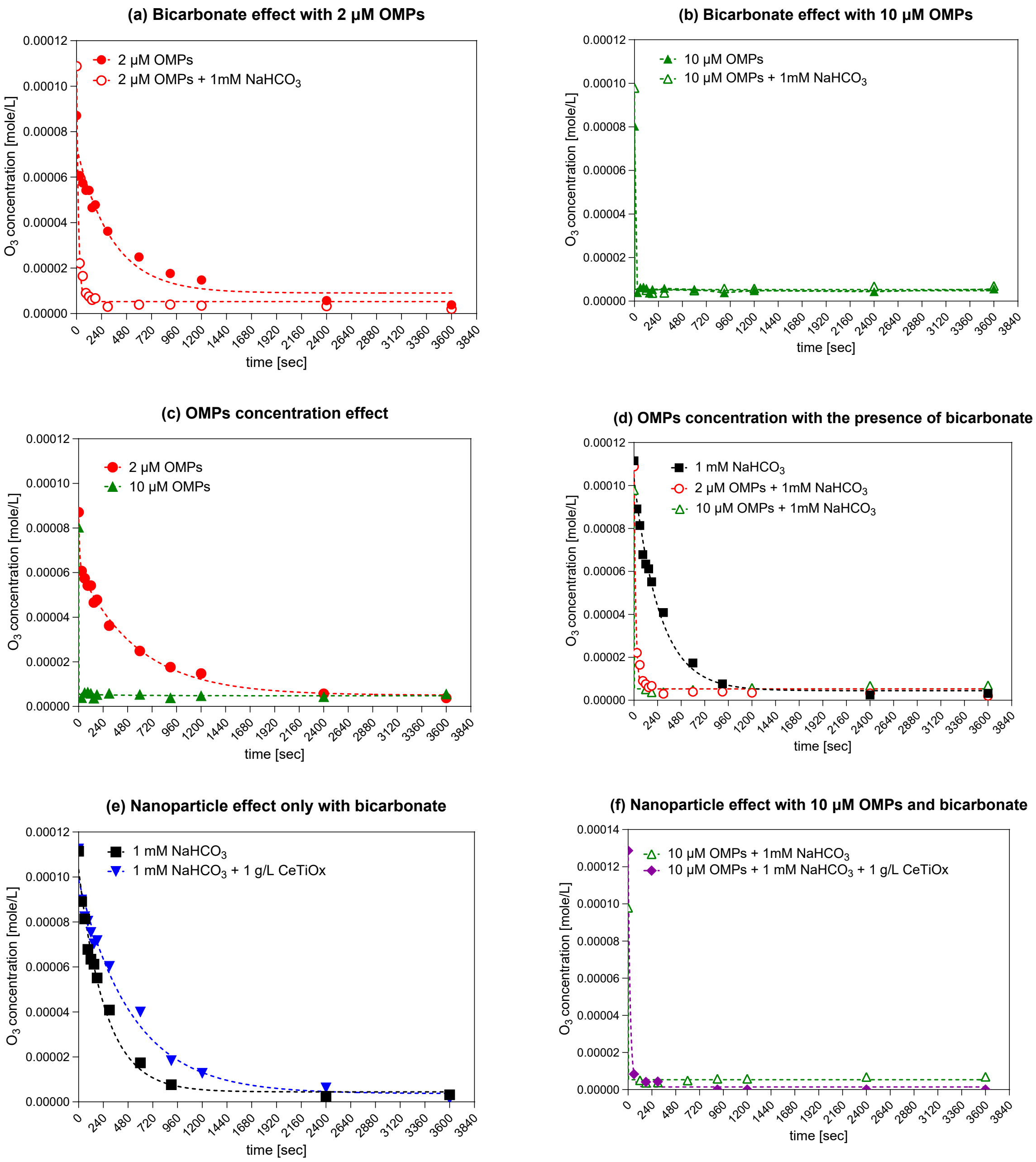

Figure S4. Degradation of ozone over time (in seconds) in different matrices for exploring the effects of OMPs' concentration, bicarbonate, and catalyst presence (TOD = 100 μM, 240 mL treated volume, [NaHCO3] = 1 mM, [OMPs] = 2 or 10 μM, catalyst concentration = 1 g^{-1})

6. Interaction plots and ANOVA (RSD)

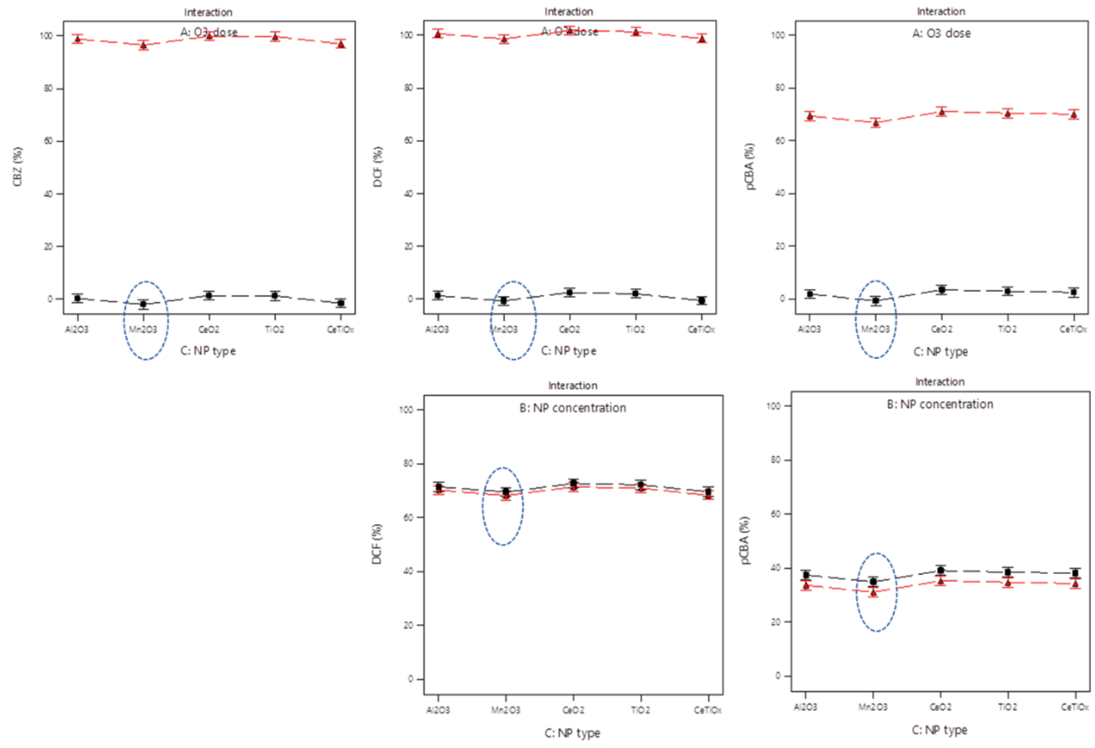

Figure S5. Interaction plots of the tested variables (NPs concentration, NPs type, and Ozone dose) for CBZ, DCF, and pCBA.

Table S4. ANOVA analysis

Source	Sum of Squares	df	Mean Square	F-value	p-value
**Carbamazepine (CBZ)**					
**Model**	1.18E+05	6	19602.64	1072.35	< 0.0001*
A-O_3 dose	1.10E+05	1	1.10E+05	6004.26	< 0.0001*
C-NP type	157.39	4	39.35	2.15	0.0829
A^2	7785.73	1	7785.73	425.91	< 0.0001*
**Residual**	1334.44	73	18.28		
**Cor Total**	1.19E+05	79			
**Diclofenac (DCF)**					
**Model**	1.20E+05	9	13307.44	807.47	< 0.0001*
A-O3 dose	1.10E+05	1	1.10E+05	6685.93	< 0.0001*
B-NP concentration	0.7808	1	0.7808	0.0474	0.8283
C-NP type	137.98	4	34.49	2.09	0.0909
A^2	9490.22	1	9490.22	575.85	< 0.0001*
B^2	39.78	1	39.78	2.41	0.1248
B^3	101.9	1	101.9	6.18	0.0153*
**Residual**	1153.63	70	16.48		
**Cor Total**	1.21E+05	79			

**Model**	71170.18	4	17792.55	491.22	< 0.0001*
A-O_3 dose	70598.2	1	70598.2	1949.08	< 0.0001*
B-NP concentration	196.79	1	196.79	5.43	0.0225*
B^2	358.54	1	358.54	9.9	0.0024*
**B^3**	387.53	1	387.53	10.7	0.0016*
**Residual**	2680.37	74	36.22		
**Cor Total**	73850.55	78			
***para*-chlorobenzoic acid (pCBA)**					
**Model**	56373.56	9	6263.73	333.73	< 0.0001*
A-O3 dose	55934.53	1	55934.53	2980.14	< 0.0001*
B-NP concentration	80.29	1	80.29	4.28	0.0423*
C-NP type	167.49	4	41.87	2.23	0.0744
A^2	237.6	1	237.6	12.66	0.0007*
B^2	189.41	1	189.41	10.09	0.0022*
B^3	124.61	1	124.61	6.64	0.0121*
**Residual**	1313.84	70	18.77		
**Cor Total**	57687.4	79			

7. Degradation of OMPs under different conditions, effect of catalyst concentration

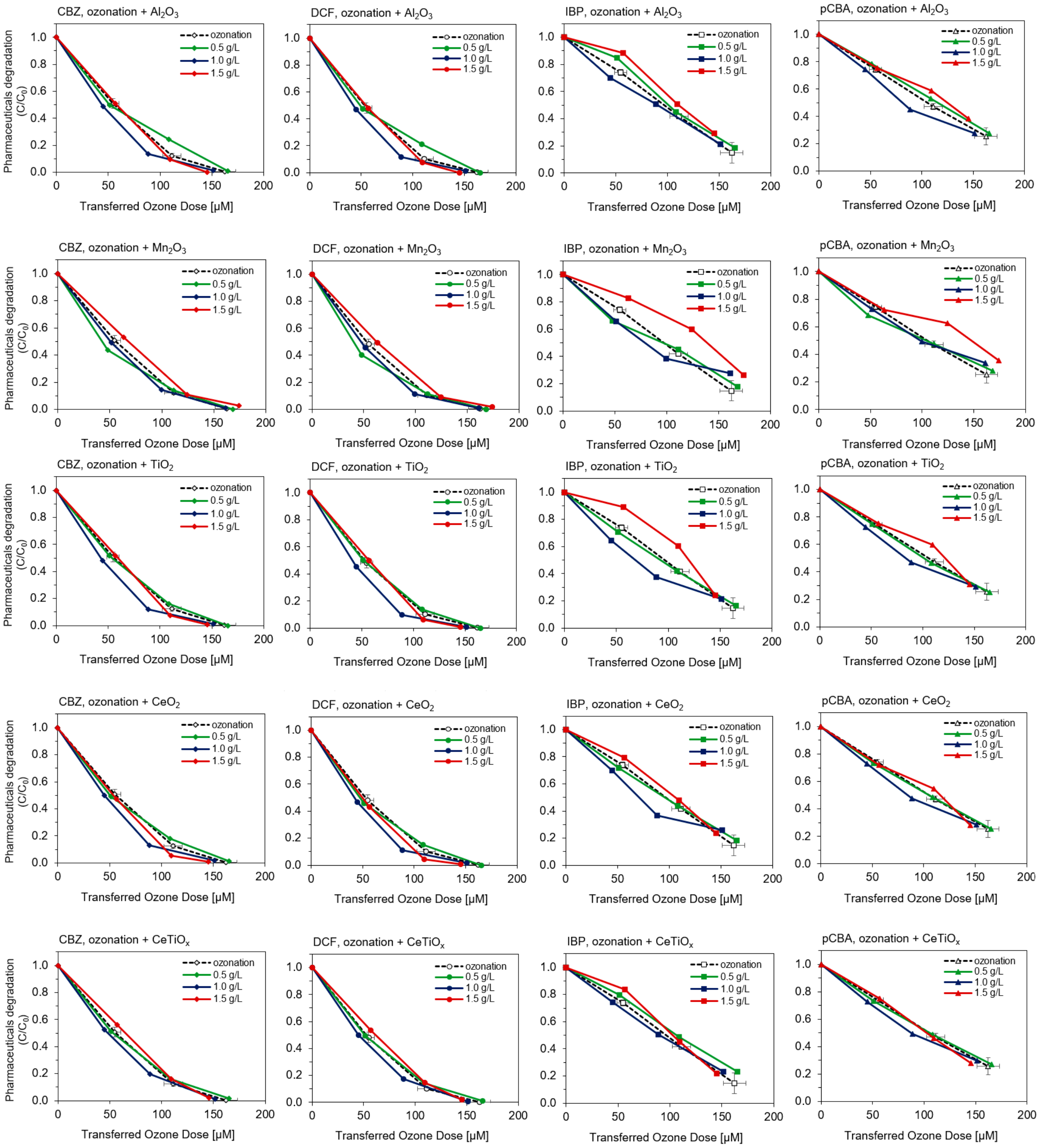

Figure S6a. Degradation of CBZ, DCF, IBP, and pCBA (starting from left to right) under three different ozone doses (50 – 150 μM) in the presence of metal oxides; *α*-Al_2O_3, Mn_2O_3, TiO_2, CeO_2, and CeTiOx (starting from top to bottom) at different concentrations; 0.5- (green), 1.0- (blue), and 1.5 (red) g L^{-1}.

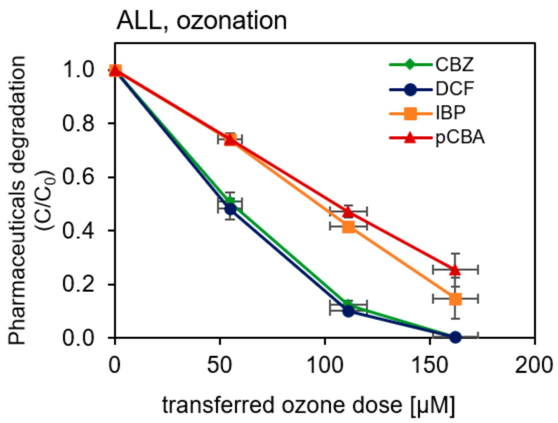

Figure S6b. Degradation of CBZ (green), DCF (blue), IBP (orange), and pCBA (red) under different ozone doses (0 – 150 μM).
